# Estrogen Regulation of mTOR Signaling and Mitochondrial Function in Invasive Lobular Carcinoma Cell Lines Requires WNT4

**DOI:** 10.3390/cancers12102931

**Published:** 2020-10-12

**Authors:** Madeleine T. Shackleford, Deviyani M. Rao, Evelyn K. Bordeaux, Hannah M. Hicks, Christina G. Towers, Joseph L. Sottnik, Steffi Oesterreich, Matthew J. Sikora

**Affiliations:** 1 Department of Pathology, University of Colorado Anschutz Medical Campus, Aurora, CO 80045, USA; madeleine.shackleford@cuanschutz.edu (M.T.S.); DEVIYANI.RAO@CUANSCHUTZ.EDU (D.M.R.); evelyn.bordeaux@cuanschutz.edu (E.K.B.); HANNAH.M.HICKS@CUANSCHUTZ.EDU (H.M.H.); JOSEPH.SOTTNIK@CUANSCHUTZ.EDU (J.L.S.); 2 Department of Pharmacology, University of Colorado Anschutz Medical Campus, Aurora, CO 80045, USA; CHRISTINA.TOWERS@CUANSCHUTZ.EDU; 3 Women’s Cancer Research Center, Dept. of Pharmacology & Chemical Biology, UPMC Hillman Cancer Center, Pittsburgh, PA 15232, USA; oesterreichs@upmc.edu

**Keywords:** WNT4, Wnt signaling, estrogen, estrogen receptor, breast cancer, lobular carcinoma, mTOR, MCL-1

## Abstract

**Simple Summary:**

Invasive lobular carcinoma (ILC) is a common but understudied breast cancer subtype. ILC is presumed to be a low-risk disease in part because nearly all ILCs contain the estrogen receptor (ER). However, we previously showed that ER has unique functions in ILC cells, including driving expression of the Wnt ligand WNT4. WNT4 signaling is required for ILC cell proliferation and survival, but the mechanisms and targets of WNT4 signaling in ILC is unknown. We found that WNT4 regulates mTOR signaling via S6 kinase, and controls levels of MCL-1 protein, ultimately regulating mitochondrial function and cellular metabolism. These findings offer new insight into a novel Wnt signaling pathway and identify new targets to inhibit WNT4 signaling as potential treatments against ILC cells.

**Abstract:**

Invasive lobular carcinoma of the breast (ILC) is strongly estrogen-driven and represents a unique context for estrogen receptor (ER) signaling. In ILC, ER controls the expression of the Wnt ligand WNT4, which is critical for endocrine response and anti-estrogen resistance. However, signaling mediated by WNT4 is cell type- and tissue-specific, and has not been explored in ILC. We utilized reverse phase protein array (RPPA) to characterize ER and WNT4-driven signaling in ILC cells and identified that WNT4 mediates downstream mTOR signaling via phosphorylation of S6 Kinase. Additionally, ER and WNT4 control levels of MCL-1, which is associated with regulation of mitochondrial function. In this context, WNT4 knockdown led to decreased ATP production and increased mitochondrial fragmentation. WNT4 regulation of both mTOR signaling and MCL-1 were also observed in anti-estrogen resistant models of ILC. We identified that high WNT4 expression is associated with similar mTOR pathway activation in ILC and serous ovarian cancer tumors, suggesting that WNT4 signaling is active in multiple tumor types. The identified downstream pathways offer insight into WNT4 signaling and represent potential targets to overcome anti-estrogen resistance for patients with ILC.

## 1. Introduction

Invasive lobular carcinoma of the breast (ILC) is the second most common histologic subtype of breast cancer [1,2,3]. Classically, ILC is characterized by small linear chain-forming neoplastic cells, which invade the mammary ducts, stroma, and adipose tissue in a characteristic single-file pattern. As such, ILC is often difficult to detect via physical exam and mammography, resulting in a delayed diagnosis and more advanced disease at presentation. Additionally, ILC is associated with unique sites of metastasis [4,5] and more challenging surgical management [6,7]. Biomarkers for ILC are consistent with the hormone-dependent “luminal A” molecular subtype (e.g., estrogen receptor alpha (ERα, hereafter ER) and progesterone receptor positive, human epidermal growth factor receptor 2 (HER2) negative)) [1,3]. About 95% of ILC tumors express ER [3] and overall appear to be exquisitely sensitive to and dependent on the steroid hormone estrogen. For example, estrogen-based hormone replacement therapy (with or without progestin) more strongly increases the incidence of ILC vs. the more common invasive ductal carcinoma (IDC) [2]. Taken together, these observations support the current paradigm that patients with ILC are ideal candidates for treatment with anti-estrogen therapies. However, retrospective studies suggest patients with ILC may not receive similar benefit from anti-estrogens as patients with IDC, i.e., poorer outcomes with adjuvant tamoxifen [8,9] and poorer long-term outcomes >5–10 years post-diagnosis [10,11,12,13]. Consistent with clinical observations, we identified tamoxifen-resistance in ER-positive ILC models, including de novo ER partial agonism by anti-estrogens [14,15], tamoxifen-resistance is driven by diverse mechanisms across ILC models [16,17,18]. Further, we recently reported that post-transcriptional regulation of ER and protein turnover in response to ligand binding is distinct in ILC cells [19]. These data suggest that ER function and signaling is unique in the context of ILC.

Studies using laboratory models of ILC and tumor profiling through The Cancer Genome Atlas (TCGA) support that aspects of cell signaling are distinct in ILC vs. IDC cells. TCGA analyses comparing luminal A ILC to luminal A IDC [3] using reverse-phase protein array (RPPA) data identified differential activity of PTEN and downstream Akt signaling. Additionally, each of the transcriptional subtypes of ILC (reactive, immune, and proliferative) showed distinct signaling features in RPPA analyses (e.g., high c-kit; high STAT5; high DNA repair protein signature, respectively). Consistent with distinct signaling contexts in ILC vs. IDC, we reported that ER regulates unique target genes in ILC cells via distinct ER DNA binding patterns [14]. These ILC-specific ER target genes mediate ILC-specific signaling pathways that are critical for endocrine response and resistance, for example, estrogen-driven regulation of *WNT4* expression and novel WNT4 signaling pathways [20,21]. However, our understanding of ER-driven signaling at the protein level in ILC cells remains limited, as studies to date either cannot define dynamic changes caused by ER activation (i.e., are from static samples as in TCGA) or are focused on the ER-driven transcriptome. Proteomic studies in ILC with estrogen or anti-estrogen treatment are needed to better understand dynamic ER-driven signaling in ILC.

We identified the Wnt ligand WNT4 as a critical signaling molecule transcriptionally induced by ER specifically in ILC cells [20]. WNT4 is unique among the Wnt protein family in its diverse cell type-specific roles, having been shown to either activate or suppress both canonical and non-canonical Wnt signaling pathways (discussed in [21]). In the normal mammary gland, WNT4 is induced by progesterone in progesterone receptor (PR) positive luminal epithelial cells, then secreted to act in a paracrine manner to activate canonical β-catenin-dependent Wnt signaling in neighboring myoepithelial cells [22,23,24,25]. In ILC cells, WNT4 regulation and signaling is “hijacked” from PR and falls under the direct control of ER [14,20], but the mechanism by which WNT4 engages downstream signaling is unclear. Hallmark genetic loss of E-cadherin (*CDH1*) in ILC cells is associated with dysfunction of catenin proteins, including destabilization and loss of β-catenin protein in cell lines and tumors, resulting in impaired canonical Wnt signaling [3,20]. Further, we recently reported that WNT4 secreted from ILC cells (and other models) is dysfunctional in paracrine activity, and that WNT4 instead activates signaling by a cell-intrinsic, intracellular mechanism [21]. Taken together, defining ER-driven and WNT4-driven signaling is necessary to both understand ILC biology and identify new target treatments for ILC.

To address these gaps in our understanding of ER function in ILC, we used RPPA analyses of ILC models to characterize ER-driven signaling in ILC and to determine the role of WNT4 in mediating ER-driven signaling. These studies identified that estrogen activates distinct protein signaling pathways in ILC cells when compared to IDC cells, including a specific component of PI3K/Akt/mTOR signaling. Within the PI3K/Akt/mTOR signaling pathway, WNT4 was required most notably for downstream mTOR activity, in addition to regulation of mitochondrial function. These observations led us to further examine the mechanisms by which ER-driven WNT4 signaling mediates cell proliferation and survival, as well as endocrine response and resistance, in ILC cells.

## 2. Results

### 2.1. Estrogen Activates a Distinct Subset of PI3K-mTOR Related Signaling in ILC Cells via WNT4

**Figure 1:**
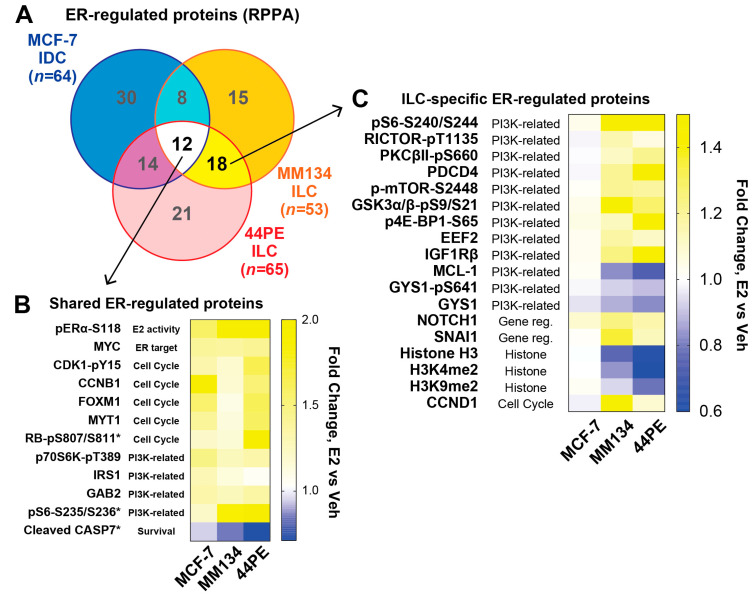
Estrogen regulates distinct protein targets in invasive lobular carcinoma (ILC) cells vs. MCF-7 invasive ductal carcinoma (IDC) cells. Reverse phase protein array (RPPA) was used to identify signaling changes after treatment with estrogen, comparing ILC models MDA MB 134VI (MM134) and SUM44PE (44PE) to IDC model MCF-7. Cells were hormone-deprived prior to treatment with 100pM estradiol (E2) for 24 h (biological triplicate). (**A**), RPPA targets with significant changes for E2 vs. vehicle (-E2) samples were compared per cell line (q < 0.05 for MM134 and 44PE; q < 0.25 for MCF-7; see text). (B–C), Heatmaps for fold change, E2 vs. veh (mean of biological triplicate). Gene ontology derived from DAVID database. (**B**), Shared/common E2-regulated targets in all three cell lines. * MCF-7 E2 vs. veh q > 0.05. (**C**), E2-regulated targets identified in both ILC models but not in MCF-7 cells.

We profiled estrogen-driven signaling by RPPA, comparing hormone-deprived cells (vehicle treated, 0.01% ethanol) vs. 100pM estradiol (E2) for 24 h. RPPA was performed with ILC cell lines MDA MB 134VI (MM134) and SUM44PE (44PE) compared to IDC cell line MCF-7 (Figure 1A). As the primary goal of this analysis was to identify ILC-specific ER targets, we used a less stringent false discovery rate for MCF-7 (FDR q < 0.25; *t*-test *p* < 0.05) than the ILC models (FDR q < 0.05) with the goal of preventing modestly ER-regulated targets in MCF-7 (i.e., with FDR q > 0.05) from being called “ILC-specific”. In all three cell lines, we identified that estrogen activated canonical ER-driven pathways, including increasing levels of MYC and cell cycle-related proteins (Figure 1B). Other shared ER targets included activation of PI3K pathway proteins (e.g., phospho-S6 Kinase/p70S6K, phospho-S6-S235/S236) and suppression of caspase 7 cleavage. These shared ER targets parallel our prior observations that ER regulates shared canonical target genes across IDC and ILC cell lines, in addition to regulating ILC-specific target genes [14]. Consistent with the latter, we identified 18 proteins regulated by ER in MM134 and 44PE, but not in MCF-7 (ILC-specific ER targets, Figure 1C). These mainly represent PI3K-related signaling (e.g., phospho-S6-S240/S244, phospho-mTOR, total MCL-1) or transcriptional control (e.g., NOTCH, SNAI1; we reported the latter previously [26]). Of note, RPPA showed that estrogen reduced histone H3 levels in ILC cells, but this is likely a subpopulation of total histone H3 [27] as the lysis buffer used for RPPA cannot solubilize histones (Figure S1, see RPPA lysis conditions in Section 4). The differential activation of PI3K-related signaling targets in MCF-7 vs. the ILC models (Figure 1C) may be related to *PIK3CA* mutational status (MCF-7 are mutant, both ILC models are wild-type [28]). However, since we previously identified WNT4 as critical for estrogen-driven proliferation in ILC cells, and non-canonical Wnt signaling has been shown to activate mTOR [29], we examined the role of WNT4 in ER signaling via the PI3K-mTOR pathway in ILC cells.

**Figure 2:**
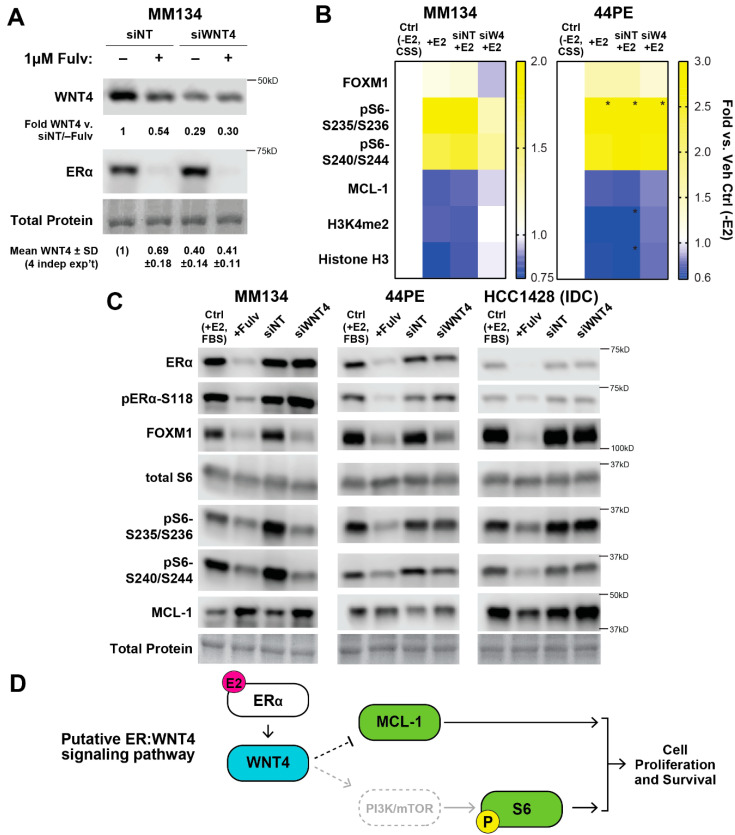
WNT4 is required for key components of estrogen receptor (ER) signaling. (**A**) MM134 cells in full serum were reverse transfected with the indicated siRNA. Then, 24 h later, cells were treated with fulvestrant, and lysates were harvested after an additional 24 h (48 h post-transfection). Total protein detected with Ponceau staining, quantification is vs. total protein. Average quantification from 4 independent experiments shown at bottom. (**B**) E2-driven RPPA signaling from Figure 1 (*n* = 18) were compared to siRNA targeting WNT4 (siWNT4) RPPA targets from Figure S2 (*n* = 52), identifying 6 targets for which siWNT4 suppressed E2-driven signaling. Mean signal of biological triplicate shown, fold vs. veh control (-E2). * value outside scale. (**C**) Targets from (B) were validated using the reciprocal treatment approach vs. RPPA analyses. Cells in complete fetal bovine serum (FBS) were treated with fulvestrant (Fulv) to block ER signaling or transfected with siRNA as indicated. Total protein by Ponceau staining, quantification in Figure S2D. (**D**) WNT4 is required for E2-mediated regulation of mTOR signaling and MCL-1 levels, which may mediate ILC cell proliferation and survival. WNT4 is required for key components of ER signaling. (**A**) MM134 cells in full serum were reverse transfected with the indicated siRNA. Then, 24 h later, cells were treated with fulvestrant, and lysates were harvested after an additional 24 h (48 h post-transfection). Total protein detected with Ponceau staining, quantification is vs. total protein. Average quantification from 4 independent experiments shown at bottom. (**B**) E2-driven RPPA signaling from Figure 1 (*n* = 18) were compared to siWNT4 RPPA targets from Figure S2 (*n* = 52), identifying 6 targets for which siWNT4 suppressed E2-driven signaling. Mean signal of biological triplicate shown, fold vs. veh control (-E2). * value outside scale. (**C**) Targets from (**B**) were validated using the reciprocal treatment approach vs. RPPA analyses. Cells in complete FBS were treated with fulvestrant (Fulv) to block ER signaling or transfected with siRNA as indicated. Total protein by Ponceau staining. (**D**), WNT4 is required for E2-mediated regulation of mTOR signaling and MCL-1 levels, which may mediate ILC cell proliferation and survival.

We previously showed that *WNT4* gene expression is directly regulated by ER [14,20] and that WNT4 is required for estrogen-mediated proliferation and survival in ILC cells [20]. However, WNT4 protein regulation has not been examined, and signaling regulated by WNT4 in ILC (via novel, cell-intrinsic Wnt signaling [21]) is yet unknown. To validate WNT4 protein regulation by ER, we confirmed (a) blocking ER with the anti-estrogen fulvestrant reduced WNT4 protein, and (b) small interfering RNA (siRNA) targeting WNT4 (siWNT4) reduced WNT4 protein (Figure 2A; we showed *WNT4* mRNA suppression and specificity of the siWNT4 pool previously [20]). Combining fulvestrant with siWNT4 did not further reduce WNT4 protein levels. We next used RPPA analyses to profile WNT4 signaling and compared hormone-deprived ILC cells treated with estrogen (as above) transfected with either non-targeting siRNA or *WNT4*-targeting siRNA (E2 + siNT vs. E2 + siWNT4, respectively). Consistent with the critical role of WNT4 in ILC cell proliferation and survival [20], siWNT4 impacted signaling related to cell cycle progression and checkpoints, and growth factor signaling via PI3K-mTOR, in MM134 and 44PE (Figure S2A–C).

We hypothesized that since WNT4 is induced by estrogen and required for ER-driven ILC phenotypes, estrogen-induced signaling blocked by siWNT4 would represent primary WNT4 targets. We compared siWNT4-mediated signaling changes to estrogen-mediated changes in ILC cells and identified six targets for which estrogen-regulated changes were ablated by WNT4 knockdown (Figure 2B). WNT4 knockdown blocked estrogen-mediated FOXM1 induction, S6 phosphorylation, and MCL-1 depletion in ILC cells (histone H3 depletion was also blocked by siWNT4). Using a reciprocal approach to the hormone-deprived setting used for RPPA analyses, we confirmed that siWNT4 suppressed ER signaling in full serum (i.e., hormone-replete conditions) and mimicked ER inhibition with fulvestrant (Figure 2C, Figure S2D). This approach confirmed that FOXM1, MCL-1, and pS6 are regulated by ER:WNT4 in MM134 and 44PE (pS6-S235/S236 was modestly regulated in 44PE, leading us to focus on S240/S244). For these targets, a similar role for ER:WNT4 was not observed in IDC model HCC1428. HCC1428 expresses high *WNT4* mRNA similar to that observed in ILC cell lines but does not depend on WNT4 for estrogen-driven proliferation or survival [20], supporting that the identified signaling targets represent ILC-specific WNT4 signaling. In hormone-depleted conditions +E2 (i.e., as in the RPPA analysis), we confirmed the observed effects of siWNT4 in MM134, and did not observe suppression of signaling by siWNT4 in IDC model MCF-7 (Figure S2E). Of note, the siNT construct caused a non-specific increase in phosphorylation of some mTOR targets (similar to our prior observations with siNT controls [20]). This effect of siNT was blocked by siWNT4 (Figure S2F), supporting that WNT4 regulates the identified targets. Taken together, these studies suggest that FOXM1, mTOR signaling, and MCL-1 are key downstream targets of WNT4 signaling in ILC cells. However, we found that WNT4 regulation of FOXM1 levels is likely an indirect effect of WNT4 control of cell cycle progression (Document S1). Based on this, we further investigated mTOR signaling and MCL-1 as putative targets of ER:WNT4 signaling (Figure 2D).

### 2.2. WNT4 Regulates mTOR Signaling Downstream of mTOR Kinase Activity

**Figure 3:**
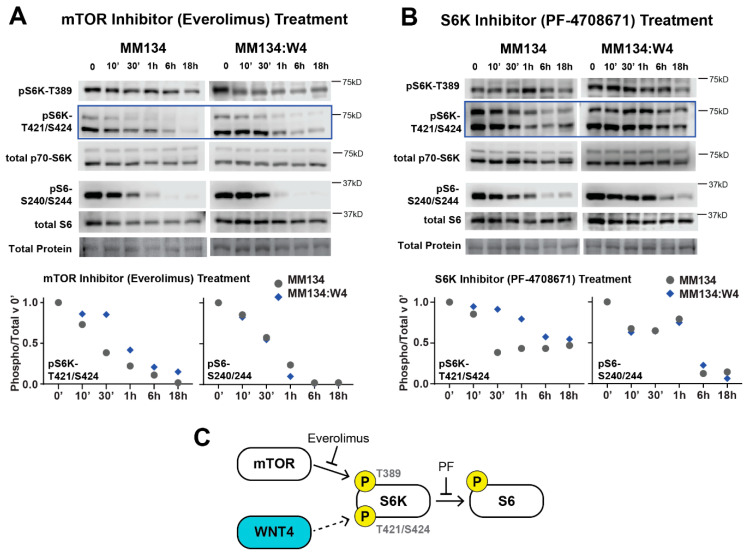
WNT4 regulates S6K phosphorylation at T421/S424. (**A**,**B**), Parental MM134 cells or WNT4-overexpressing cells (MM134:W4) in full serum were treated with 10 nM everolimus (**A**) or 30 μM PF-4708671 (**B**) for increasing time as indicated. Blue boxes highlight time-dependent shifts in kinase inhibitor response driven by WNT4 over-expression (discussed in text). pS6K-T421/S424 and pS6 levels are quantified at bottom (normalized to total S6K or S6, respectively); data are representative of 2–3 replicate experiments. Total protein detected with Ponceau staining. (**C**) WNT4 regulates phosphorylation of S6K at T421/S424, and as such required for S6K activity, but S6K activity remains dependent upon upstream activation by mTOR (T389).

We next examined the regulation of mTOR signaling by ER:WNT4 signaling. RPPA showed that siWNT4 reduced phosphorylation of S6 but not other E2-induced mTOR targets. We confirmed by immunoblot that siWNT4 suppressed S6 phosphorylation, but not mTOR (S2448) or S6 Kinase (S6K, T389) phosphorylation (Figure S3). However, in further examining S6K phosphorylation sites, we found that WNT4 knockdown suppressed S6K phosphorylation at T421/S424 (Figure S3). This suggests WNT4 plays a specific role in releasing S6K from its pseudo-substrate inhibitory loop, thus regulating S6K activity via a mechanism distinct from mTOR [30]. Based on this, we hypothesized that WNT4 may promote S6K activity during mTOR inhibition, and assessed signaling responses to the mTOR inhibitor everolimus or S6K inhibitor PF-4708671 (PF, [31]) in WNT4 over-expressing MM134 cells (MM134:W4; described previously [21]). WNT4 over-expression modestly delayed the loss of S6K phosphorylation at T421/S424 caused by everolimus (Figure 3A) or S6K inhibition (Figure 3B), but both inhibitors ultimately suppressed downstream mTOR signaling (i.e., S6 phosphorylation) in both parental and MM134:W4 cells. These data suggest WNT4 is necessary for S6K activity via phosphorylation at T421/S424 but is not sufficient to drive activity without active upstream mTOR kinase activity or with direct S6K inhibition (Figure 3C).

### 2.3. ER:WNT4 Regulation of MCL-1 Is Associated with Metabolic Dysregulation

The anti-apoptotic BCL2-family protein MCL-1 [32] was also identified in our RPPA analyses as an ER:WNT4 target (Figure 2B,D), estrogen reduced MCL-1 protein levels, and this was reversed by WNT4 knockdown. Notably, this reduction in MCL-1 levels by ER in ILC cells contrasts ER regulation of MCL-1 in IDC cells, where ER activation increases MCL-1 levels and promotes cell survival [33], suggesting a distinct role for ER control of MCL-1 in ILC cells. To confirm this, we examined whether MCL-1 levels contributed to control of intrinsic apoptosis. We treated ILC cells with fulvestrant (to increase MCL-1 levels) and/or MCL-1-specific inhibitor A-1210477 in combination with other BH3 mimetics (inhibitors of anti-apoptotic Bcl-2-family proteins). Though blocking MCL-1 modestly sensitized cells to other BH3 mimetics, fulvestrant caused no shifts in sensitivity to any tested combination of BH3 mimetics (Figure S4A). Taken together, ER:WNT4 regulation of MCL-1 is not directly linked to the role of MCL-1 in intrinsic apoptosis.

**Figure 4:**
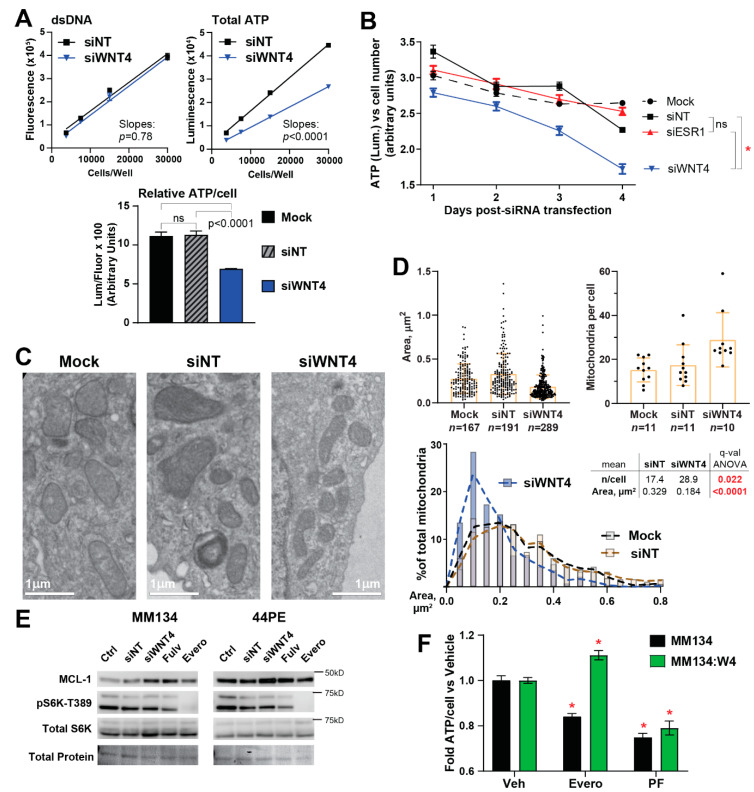
WNT4 knockdown compromises cellular metabolism and drives mitochondrial fragmentation. (**A**) MM134 cells were transfected with siRNA as indicated, 48 h later cells were harvested and re-plated in a two-fold serial dilution in technical quadruplicate. Then, 24 h later (72 h post-transfection) dsDNA and total ATP were quantified from separate samples. Bar graph at bottom represents 30 k/well data from standard curves (mock excluded from standard curves for clarity). Comparisons with ANOVA + Tukey correction. (**B**) MM134 cells transfected as indicated, with dsDNA and total ATP quantified at the indicated time from matched samples. Cell number was determined by interpolating dsDNA fluorescence vs. a standard curve, and total ATP (luminescence) was normalized vs. cell number. Comparisons by two-way ANOVA; α = 0.01. * *p* < 0.0001. (**C**) MM134 cells were fixed for transmission electron microscopy 72 h post-transfection. Representative images of mitochondrial morphology shown. (**D**), Mitochondrial dimensions were quantified from TEM images using ImageJ (see Section 4). Box-and-whiskers represent mean ± SD. Histogram is derived from area data shown as dot plot at top. (**E**) Cells in full serum were either reverse transfected with the indicated siRNA for 48 h, or treated with 100 nM fulvestrant, or 100 nM everolimus for 24 h. S6K phosphorylation (mTOR target site T389) shown as positive control for mTOR inhibition by everolimus. Total protein detected with Ponceau staining. (**F**) MM134 were treated with 100 nM everolimus or 30 µM PF-4708671 for 72 h; ATP and dsDNA were quantified as above. * q < 0.05 vs. vehicle by two-way ANOVA.

MCL-1 also plays a critical role in mitochondrial dynamics, regulating the balance of mitochondrial fission vs. fusion and, subsequently, cellular capacity for oxidative phosphorylation [34,35,36,37,38,39,40]. Based on this, we hypothesized that ER:WNT4 control of MCL-1 indicates a role for WNT4 in mitochondrial dynamics and cellular metabolism in ILC cells, and found siWNT4 caused a decrease in total ATP content per cell (Figure 4A). The decrease in cellular ATP levels was progressive over a 1–4d time course after siWNT4 (Figure 4B). ER knockdown did not produce a similar decrease in ATP levels, which parallels our prior observations that WNT4 knockdown, but not ER inhibition, induces cell death in ILC cells [20]. This suggests that continued ER signaling may allow mitochondrial dysfunction to manifest upon WNT4 knockdown, while suppressing ER reduces cell growth and metabolic demand, effectively protecting cells from loss of ER-driven WNT4 expression.

The decreased ATP levels and increased MCL-1 observed after WNT4 knockdown is consistent with mitochondrial fission or fragmentation [37,40], so we used transmission electron microscopy to investigate mitochondrial phenotype after WNT4 knockdown. At 72 h post-transfection (i.e., during progressive loss of ATP production, Figure 4A,B), cells with siWNT4 had an increased number of smaller mitochondria (Figure 4C). Cells with control siRNA had a mean (±SD) of 17.4 ± 9.24 mitochondria per cell with a mean area of 0.329 ± 0.233 μm^2^, siWNT4 caused a >65% increase in mitochondria per cell (mean = 28.9 ± 12.3) while decreasing area by ~45% (mean = 0.184 ± 0.135 μm^2^) (Figure 4D). This supports that WNT4 knockdown drives mitochondrial fission or fragmentation. Taken together, regulation of MCL-1 levels is linked to a critical role for ER:WNT4 signaling in mitochondrial function and cellular metabolism.

Mitochondrial function and cellular metabolism are also regulated by mTOR [39,41], and based on this, we investigated how ER:WNT4 control of mTOR/S6K impacted cellular metabolism in ILC cells. Notably, in ILC cells, ER:WNT4 control of mTOR/S6K is likely independent of control of MCL-1 levels. In IDC cells, ER-driven mTOR activity increases MCL-1 levels [33] and mTOR inhibition reduces MCL-1 [42]. Conversely, ER suppresses MCL-1 protein levels in ILC (Figure 2; *MCL1* mRNA expression is not ER-regulated, Figure S4B) and inhibiting mTOR with everolimus did not reduce MCL-1 levels in MM134 or 44PE cells (Figure 4E). We further examined whether ER:WNT4 regulated mTOR signaling controlled cellular metabolism, and found that treatment of MM134 cells with either everolimus or PF-4708671 (S6K inhibitor) decreased cellular ATP levels (Figure 4F). Importantly, WNT4 over-expression rescued the effects of everolimus, but not S6K inhibition, consistent with WNT4 regulating mTOR signaling downstream of mTOR via S6K. These data suggest mTOR/S6K and MCL-1 are independent targets of WNT4 signaling that converge on regulation of mitochondrial function and cellular metabolism.

### 2.4. WNT4 Signaling Remains Active during Anti-Estrogen Resistance in ILC Cells

We previously reported that long-term estrogen deprived (LTED; mimicking aromatase inhibitor-resistance) variants of ILC cell lines remain WNT4-dependent [20] and hypothesized that WNT4-driven pathways identified in the parental cells would also be active in LTED models. LTED models 44:LTED/A and 134:LTED/E (derived from 44PE and MM134, respectively [20]) were analyzed by RPPA and compared to hormone-deprived parental cells to identify signaling adaptations during LTED. LTED cells were also transfected with siNT or siWNT4 as above to profile WNT4-driven signaling during LTED and determine whether WNT4 signaling identified in parental cells was re-activated in LTED models despite loss of estrogen-mediated ER regulation of *WNT4*.

**Figure 5:**
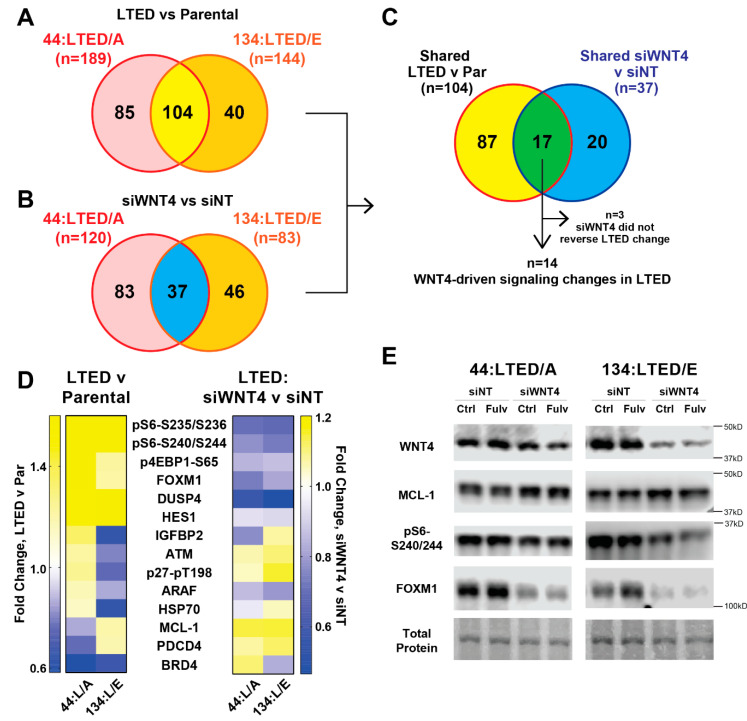
ER:WNT4 signaling targets are similarly active in anti-estrogen resistant ILC models. (**A**) Long-term estrogen deprived (LTED) cells were compared to their hormone-deprived (vehicle treated) parental cell line; differences at q < 0.05 shown. (**B**) LTED cells were reverse transfected with the indicated siRNA for 48 h prior to harvest for RPPA analyses. RPPA protein signaling changes for siWNT4 + E2 vs. siNT + E2 shown (*p* < 0.05). (**C**) Overlap between signaling activation in LTED (from (**A**)) vs. WNT4-driven signaling (from (**B**)) to identify specific WNT4-driven signaling that is activated during anti-estrogen resistance. For *n* = 3, siWNT4 did not reverse the changes seen in LTED v parental in at least one of the two model systems. (**D**) Heatmap of *n* = 14 genes from (**C**) for LTED v parental fold change (left) and siWNT4 v siNT fold change (right). Boxes show mean of biological triplicate samples. (**E**) LTED cells (in hormone-deprivation) were reverse transfected with the indicated siRNA. Then, 24 h later, cells were treated with 1 μM fulvestrant or vehicle, and lysates were harvested after an additional 24 h. Total protein detected with Ponceau staining (also see quantification in Figure S6B).

The majority of RPPA targets altered in the anti-estrogen resistant phenotype (i.e., LTED vs. parental comparisons) were shared between the 134:LTED/E and 44:LTED/A models (Figure 5A, Figure S5A). This included fatty acid synthase (FASN) upregulation, confirming the upregulation of *FASN* and lipid metabolism genes in these models that we recently reported (Figure S5B) [43]. RPPA also confirmed changes specific to 44:LTED/A (increased ER and OCT4) vs. 134:LTED/E (decreased ER, increased phospho-NFκB), which we previously reported (Figure S5B) [20]. Comparing protein changes in LTED models vs. those caused by WNT4 knockdown identified 14 protein changes induced during LTED that are mediated by WNT4 signaling (Figure 5B–D). WNT4-driven signaling in LTED models included key WNT4 targets identified in parental cells, e.g., S6 and MCL-1, which we confirmed by immunoblotting (Figure 5E). siWNT4 suppressed S6 phosphorylation (S240/S244) and increased MCL1 levels as observed in parental cells. These WNT4-driven signaling changes were also confirmed in additional ILC:LTED cell lines (Figure S6A,B). These data support that WNT4 signaling is active in anti-estrogen resistant LTED models (i.e., in an estrogen-independent context), and that WNT4 remains required for regulating key downstream pathways (mTOR signaling and MCL-1) in the LTED setting.

### 2.5. WNT4 Signaling Functions in ILC Tumors and Serous Ovarian Cancer

**Figure 6:**
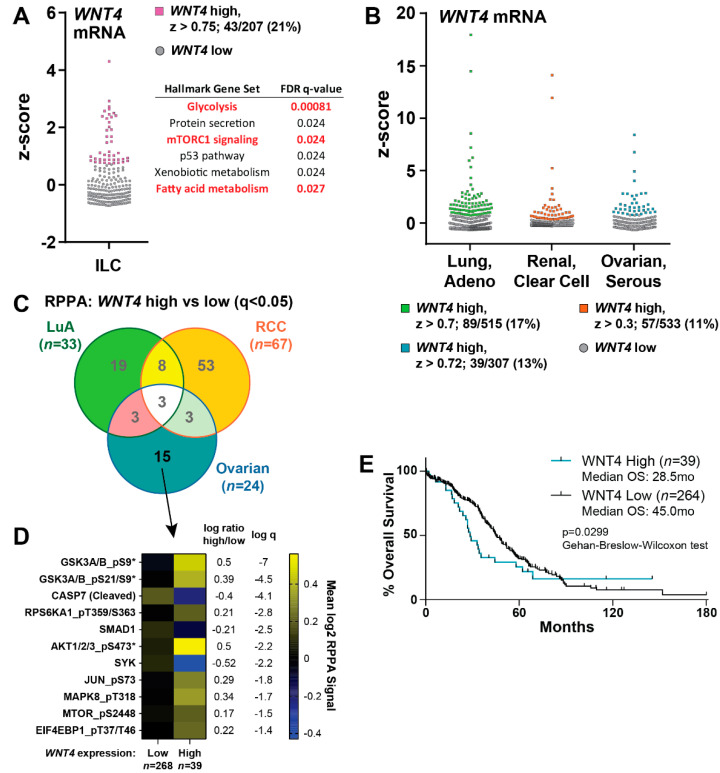
High WNT4 expression is associated with activated mTOR signaling in ILC and serous ovarian cancer. (**A**) RNAseq z-scores used to identify tumors with high WNT4 mRNA levels from The Cancer Genome Atlas (TCGA) data. Points represent individual tumor samples; colored v gray points represent individual tumor samples with high/low WNT4. *n* = 354 genes differentially expressed in WNT4-high vs. -low tumors were analyzed against the MSigDB “Hallmark” genesets. (**B**) RNAseq z-scores were used to identify WNT4-high vs. -low tumors as in (**A**). (**C**) cBio “Enrichment” tool was used to identify differences in RPPA signals for WNT4-high vs. -low tumors (q < 0.05). (**D**), Mean log2 RPPA signal for WNT4-associated RPPA targets specific to OvCa. *, GSK3A/B and AKT1/2/3 are counted as individual targets in cBio but do not have unique data (*n* = 15 from Venn yields *n* = 11 unique targets). (**E**), Overall survival data derived from cBio “Survival” tool. High v low WNT4 as in (**B**).

To examine WNT4-driven signaling in tumor tissues, we explored data from The Cancer Genome Atlas (TCGA) using the cBio Portal [44]. In ILC tumors, high *WNT4* expression was associated with gene signatures for mTORC1 signaling and cellular metabolism (*WNT4*-high *n* = 43; total *n* = 207; Figure 6A), consistent with our signaling studies. We examined whether similar signaling pathways were associated with high *WNT4* in RPPA data, but none reached statistical significance (FDR q < 0.1) likely due to limited sample size of ILC with RPPA data (total *n* = 161, *WNT4*-high *n* = 29). We expanded our RPPA analyses across other tumors from tissues that require WNT4 in development (e.g., kidney, adrenal, lung, ovary, uterus [45,46,47,48]). Renal clear cell carcinoma (RCC; total *n* = 515, *WNT4*-high *n* = 89), lung adenocarcinoma (LuA; total *n* = 533, *WNT4*-high *n* = 57), and serous ovarian cancer (OvCa; total *n* = 307, *WNT4*-high *n* = 39) presented differences in protein signaling by RPPA in *WNT4*-high tumors (Figure 6B–D, Table S1). Shared signaling changes across tumor types were limited (Figure 6C). However, β-catenin regulation was observed in LuA and RCC but not OvCa, suggesting WNT4 signaling is β-catenin-dependent vs. β-catenin-independent in LuA and RCC vs. OvCa, respectively. Since WNT4 signaling in ILC is β-catenin-independent [20,21], we focused on OvCa-specific signaling associated with high *WNT4*. This identified increased phosphorylation of PI3K/mTOR pathway proteins specifically in *WNT4*-high OvCa (Figure 6D, MCL-1 data are not available in these datasets). Phospho-S6 (S240/244) and phospho-S6K (T389) were also elevated in *WNT4*-high OvCa but did not reach statistical significance (pS6K-T421/S424 is not available by RPPA). These data in OvCa parallel our observations in ILC that WNT4 is associated with active PI3K/mTOR signaling. High *WNT4* expression in OvCa was also associated with shorter overall survival (median OS, 28.5mo v 45.0mo; Figure 6E). These data suggest that WNT4 signaling via the PI3K/mTOR pathway is active in multiple tumor types, including ILC and OvCa.

## 3. Discussion

**Figure 7:**
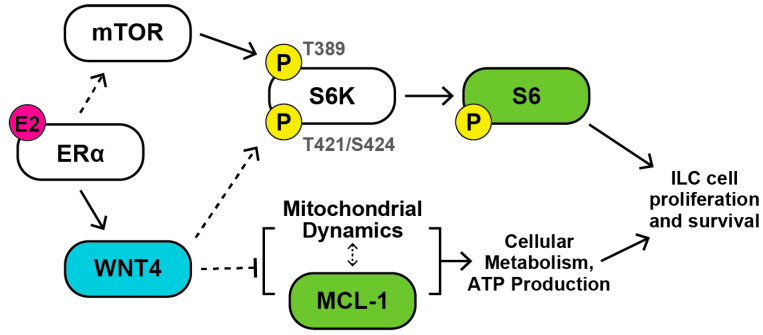
mTOR signaling and MCL-1 are key downstream targets of WNT4 in ILC cells. Our data identified mTOR signaling and mitochondrial function/dynamics as key processes downstream of WNT4. In mTOR signaling, WNT4 regulated S6K-mediated S6 phosphorylation via regulation of S6K phosphorylation at the psuedo-substrate inhibition site (T421/S424). Upstream mTOR-driven phosphorylation of S6K (e.g., at T389) was still necessary for S6K activity. WNT4 also regulated MCL-1 protein levels, which was associated with decreased mitochondrial function and increased mitochondrial fission upon WNT4 knockdown. Future studies must examine whether MCL-1 is a driver or an output of WNT4-mediated changes in mitochondrial dynamics. These two pathways are likely central to the role of WNT4 in mediating ER-driven proliferation and survival in ILC cells.

Clinical and laboratory data support that ILC represents a unique context for estrogen receptor signaling, which may be mediated in part by ER-induced *WNT4* expression. The Wnt ligand WNT4 plays a critical role in the normal mammary gland development, and similarly is required for endocrine response and resistance in ILC cells [20]. However, the mechanisms by which WNT4 mediates proliferation and survival in ILC cells are unclear, as WNT4 in ILC creates a novel context for Wnt signaling. WNT4 regulates myriad tissue-specific pathways, but β-catenin-dependent signaling is dysfunctional in ILC, and further, we found WNT4 has unique activity as an intracellular signaling molecule [3,20,21]. To address this, we used RPPA analyses to profile ER-driven signaling in ILC cells and to identify ER-driven signaling that requires WNT4 (i.e., ER:WNT4 signaling). Our study found that in ILC cells, ER:WNT4 signaling mediates downstream activity of mTOR signaling via p70-S6K, as WNT4 is required for p70-S6K phosphorylation (T421/S424) and downstream S6 phosphorylation. We also identified that ER:WNT4 signaling suppresses total MCL-1 levels, which was not associated with differential sensitivity to pro-apoptotic dugs, but instead with metabolic dysfunction and mitochondrial fragmentation upon WNT4 knockdown. Parallel signaling mediated by WNT4 was identified in anti-estrogen-resistant models of ILC. Related signaling pathways were similarly linked to *WNT4* over-expression in ILC and in serous ovarian cancer tumors. These data provide new insight into a poorly understood Wnt signaling pathway and identify signaling pathways downstream of atypical intracellular WNT4 signaling [21]. These downstream pathways (Figure 7) may be targetable to inhibit cell proliferation and survival mediated by WNT4.

Our data shows that ER drives mTOR signaling in ILC cells, with WNT4 serving as a necessary signaling partner for downstream signaling. Interestingly, rather than regulating phosphorylation of S6K at T389, we found WNT4 instead regulates phosphorylation at T421/S424. T421/S424 are part of a cluster of phosphorylation sites in the C-terminal auto-inhibitory domain of p70-S6K (pseudo-substrate inhibition), and their phosphorylation is necessary for S6K activation [30]. The regulation of phosphorylation at these sites is not fully understood, but kinases including ERK1/2 [49,50], CDK5 [51,52], JNK [53], and TBK1 [54] have been shown to target these auto-inhibitory domain sites. Neither JNK nor ERK1/2 were regulated by WNT4 knockdown in our RPPA, and we previously showed JNK is not a target of non-canonical paracrine WNT4 signaling in MM134 cells [21]. CDK5 may be a potential link between WNT4 and p70-S6K, as CDK5 has been linked to non-canonical Wnt signaling [55] as well as mitochondrial function in breast cancer cells [56]. CDK5 or other kinases (or phosphatases) may represent key steps linking WNT4 downstream to p70-S6K and defining the mechanism by which WNT4 initiates and propagates signaling is an important future direction.

WNT4 signaling is a novel regulatory mechanism for PI3K signaling via mTOR/p70-S6K. Activation of PI3K/Akt/mTOR signaling has been identified as a major feature of ILC, including increased pathway activity vs. matched IDC tumors, in three large-scale genomic analyses of ILC (The Cancer Genome Atlas, RATHER, Desmedt et al., European cohort) [3,57,58]. These observations highlight the need to better understand the PI3K-mTOR pathway in ILC. A recent study by Teo et al., linked loss of E-cadherin (i.e., the hallmark feature of ILC [3]) to Akt activation and signaling [59], E-cadherin loss has also been linked to activation of growth factor signaling via membrane receptors [60,61]. TCGA analyses also suggested that subsets of ILC may utilize distinct modes of PI3K-mTOR signaling. Among luminal A ILC, TCGA RPPA identified differential levels and phosphorylation of PI3K/Akt/mTOR pathway proteins in mRNA-driven ILC subtypes (e.g., low p70S6K and Raptor in reactive, high phospho-PRAS40 and phospho-mTOR in immune, proliferative lacked distinct differences in this pathway) [3]. Another important context for PI3K-pathway signaling in ILC is the tumor microenvironment and metastasis. ILC is likely have a distinct interaction with the immune microenvironment compared to IDC [62,63]. Additionally, Tasdemir et al., reported that in ultra-low attachment conditions (i.e., requiring anchorage-independence, mimicking metastasis), ILC cell lines were uniquely able to sustain PI3K pathway activation vs. IDC cell lines [64]. Further mechanistic studies are needed to link biomarkers with PI3K/Akt/mTOR signaling activity to identify precision targets for therapy, i.e., to identify the ideal targeted inhibitor for an individual tumor. Importantly, in considering differences in how estrogen drives PI3K/Akt/mTOR signaling in ILC vs. IDC, our RPPA studies included two ILC models (MM134, 44PE) and one IDC model (MCF-7). Proteomics analyses with more models [15,64], accounting for pathway mutations, are needed to understand these signaling pathways.

ER:WNT4 regulation of MCL-1 and mitochondrial function suggest that WNT4 is critical for cellular metabolism in ILC cells. We observed that WNT4 knockdown leads to impaired ATP production and mitochondrial fragmentation, and both of these phenotypes precede the induction of cell death (~4d post-siWNT4 transfection [20]). This suggests that WNT4-mediated control of metabolism and/or mitochondrial function are critical to ILC cell survival. Wnt signaling (both β-catenin-dependent and -independent signaling) has been previously linked to these processes [65,66,67], but direct roles for WNT4 in mitochondrial function have not previously described. However, one recent study demonstrated that Wnt4 over-expression could rescue a defect in mitochondrial function and dynamics caused by deletion of PTEN-inducible kinase 1 (PINK1) in Drosophila [68]. Though the mechanism of rescue is unclear, Wnt4 over-expression rescued flight defects in the PINK1 knockout flies, increasing ATP production and restoring mitochondria membrane potential in flight muscles. Our observations of altered mitochondrial fission, mTOR and MCL-1 regulation, and a putative role in PINK1 signaling suggest that WNT4 may have a role in regulating autophagy or specifically mitochondrial mitophagy (mitophagy). Fragmented mitochondria have impaired oxidative phosphorylation [40,69], and can ultimately undergo mitophagy with mTOR inactivated [70], this is overall consistent with the phenotype we observe upon WNT4 knockdown. PINK1 is a key mitophagy regulator [68], and the ability of WNT4 to rescue PINK dysfunction further supports a potential role for WNT4 in regulating autophagy/mitophagy. Future studies on WNT4 regulation of autophagy may provide further mechanistic insight into novel functions of WNT4 and identify unique metabolic vulnerabilities as a treatment approach for WNT4-driven cancers including ILC.

The similar signaling pathways associated with WNT4 in ILC and OvCa may parallel the critical role of WNT4 in both tissues of origin, and related tumor biology. In addition to being required for mammary gland development, WNT4 is required for the development of the ovary and Mullerian tissues [45,71,72]. Accordingly, WNT4 dysfunction is linked to a range of endocrine and gynecologic pathologies, including endometriosis [73], uterine fibroids [74], and ovarian cancer [75]. For ovarian cancer that originates from fallopian tube epithelium (FTE), transformed FTE cells must migrate to and invade the ovary to establish a tumor. WNT4 is required for this cell migration and ovary invasion in murine PTEN-null models of FTE-derived ovarian cancer [76], suggesting WNT4 is critical in early ovarian tumorigenesis. Perhaps consistent with this role of WNT4 in migration/invasion, both OvCa and ILC metastasize to the abdomen/peritoneal cavity, ILC being unique among breast cancers in this regard [4,5]. We also recently reported that *WNT4* gene expression is elevated in OvCa tumors after neoadjuvant chemotherapy [77] and taken together with the association of high WNT4 with poor overall survival, WNT4 may mediate cell survival and chemo-resistance in OvCa. Further understanding of WNT4 signaling may provide new insight into targeting metastatic and chemo-resistant OvCa and ILC.

WNT4 signaling is a key mediator of endocrine response and resistance in ILC but given the dysfunction of β-catenin-dependent signaling in ILC and novel functions of WNT4 as an intracellular signaling molecule, WNT4 signaling pathways in ILC must be identified. In this study, our RPPA analyses identified mTOR signaling and MCL-1/mitochondrial function as two key downstream effectors of WNT4 in ILC. Parallel pathways associated with WNT4 were also identified in serous ovarian cancer, suggesting that WNT4 signaling is important in multiple tumor types. Future studies will determine how WNT4 is mechanistically linked to these targets, including identification of the WNT4 “receptor” in ILC cells. Furthering our understanding of WNT4 signaling will improve opportunities for precision treatment approaches by targeting WNT4 signaling for patients with ILC, or OvCa, and support the development of strategies to overcome anti-estrogen resistance for patients with ILC.

## 4. Materials and Methods

### 4.1. Cell Culture

MDA MB 134VI (MM134) and SUM44PE (44PE) were maintained as described [14]. MCF-7 and HCC1428 were maintained in DMEM/F12 (Corning Life Sciences, Corning, NY, USA; cat#10092CV) supplemented with 10% fetal bovine serum (FBS; Nucleus Biologics, San Diego, CA, USA; cat#FBS1824). Hormone-deprivation was performed as described [78] with phenol red-free reagents in IMEM (Gibco/ThermoFisher, Waltham, MA, USA; cat#A10488) supplemented with 10% charcoal-stripped fetal bovine serum (CSS, prepared as described [78] with the same FBS as above). Parental cell lines used herein are ERα/*ESR1*-positive, and express low levels of *ESR2* and *GPER1* [79]. WNT4 over-expressing models were previously described [21] and cultured in the same conditions as parental cell lines. Long-term estrogen deprived (LTED) model establishment and culture conditions were previously described [20]. All lines were incubated at 37 °C in 5% CO_2_. Cell lines are authenticated annually via the University of Arizona Genetics Core cell line authentication service and confirmed to be mycoplasma negative every four months. Authenticated cells were in continuous culture <6 months.

17β-Estradiol (E2; cat#2824) and fulvestrant (fulv/ICI182,780; cat#1047) were obtained from Tocris Bioscience (Bio-Techne, Minneapolis, MN, USA) and dissolved in ethanol. Everolimus (evero; cat#11597), PF-4708671 (PF; cat#15018), ABT199 (cat#16233), ABT263 (cat#11500), WEHI539 (WEHI; cat#21478), and A1210477 (A121; cat#21113) were obtained from Cayman Chemical (Ann Arbor, MI, USA) and dissolved in DMSO.

### 4.2. RNA Interference

siRNAs were reverse transfected using RNAiMAX (ThermoFisher) according to the manufacturer’s instructions. All constructs are siGENOME SMARTpool siRNAs (Dharmacon/Horizon Discovery, Lafayette, CO, USA), nontargeting pool #2 (D-001206-14-05), human *WNT4* (M-008659-03-0005), human *MCL1* (M-004501-08-0005). Details regarding validation of the specific effects of the *WNT4* siRNA pool are previously described [20].

### 4.3. Reverse Phase Protein Array (RPPA)

Cells were hormone-deprived prior to reverse transfection with 10 nM siRNA as described above. Then, 24 h post-transfection, cells were treated with vehicle (0.01% EtOH) or 100 pM E2 and harvested 24 h later (48 h post-transfection, 24 h post-treatment). Cells were lysed according to core facility instructions (see below, [80]). Briefly, cells were washed twice with cold phosphate-buffered saline, and incubated on ice with 60 μL of RPPA lysis buffer (see below) for 20′. Lysates were collected by scraping and centrifuged at ~16,000× *g* at 4 °C for 10′. Supernatant was collected and utilized for RPPA analyses. RPPA lysis buffer was made with 1% Triton X-100, 50 mM HEPES (pH 7.4), 150 mM NaCl, 1.5 mM MgCl_2_, 1 mM EGTA, and 10% glycerol, supplemented with Halt protease and phosphatase inhibitor cocktail (Pierce/ThermoFisher, cat#78440).

RPPA analyses and data normalization were carried out by the MD Anderson Cancer Center Functional Proteomics Core Facility (August 2016, platform included 305 antibodies listed in Table S2). Details for RPPA signal normalization and quality control provided by the core facility are in Document S2, normalized linear data are presented in all analyses herein. Multiple testing correction was applied using the Benjamini–Hochberg method. Raw/normalized RPPA data are available in File S1, and treatment comparisons will be deposited to an Open Science Framework page upon publication [81] or will be provided upon request.

### 4.4. Immunoblotting

Whole-cell lysates were obtained by incubating cells in RPPA lysis buffer (above) for 30′ on ice. Cells were centrifuged at ~16,000× *g* for 15 m at 4 °C and the resulting supernatant was collected for analysis. Protein concentrations were measured and normalized using the Pierce BCA protein assay kit (#23225). Protein loading was kept consistent by mass across matched experiments, and standard methods were used to perform SDS-PAGE. Proteins were transferred onto PVDF membranes. Antibodies were used according to manufacturer’s recommendations: WNT4 (R&D, MAB4751, cat# 55025, also see specific immunoblot considerations [21]); ERα (Leica, Buffalo Grove, IL, USA; 6F11, cat# ER-6F11-L-F); pER-S118 (Cell Signaling, Danvers, MA, USA; 16J4, cat#2511); FOXM1 (Cell Signaling, D12D5, cat#5436); total S6 (Cell Signaling, 54D2, cat#2317); pS6-S235/236 (Cell Signaling, cat#2211); pS6-S240/244 (Cell Signaling, cat#2215); p70-S6K (Cell Signaling, 49D7, cat#2708); p-p70-S6K-T389 (Cell Signaling, 108D2, cat#9234); p-p70-S6K-T421/S424 (Cell Signaling, cat#9204); total mTOR (Cell Signaling, 7C10, cat#2983); p-mTOR-S2448 (Cell Signaling, D9C2, cat#5536); MCL-1 (Cell Signaling, D35A5, cat#5453); 4E-BP1 (Cell Signaling, cat#9452); p4E-BP1-S65 (Cell Signaling, 174A9, cat#9456); histone H3 (Abcam, Cambridge, MA, USA; cat#ab1791). Secondary antibodies were used according to manufacturer’s instruction and were obtained from Jackson ImmunoResearch Laboratories (West Grove, PA, USA), goat anti-mouse IgG (cat# 115-035-068), goat anti-rabbit IgG (cat# 111-035-045) and goat anti-rat IgG (cat# 112-035-062). Chemiluminescence was used to detect antibodies and either film or the LICOR c-Digit (LI-COR Biosciences, Lincoln, NE, USA) was used to develop the immunoblots. Total protein (Ponceau) served as a loading control. Representative immunoblots with size markers indicated are shown in Document S3.

### 4.5. Cell Proliferation

Total double-stranded DNA was measured as a surrogate for total cell number by hypotonic lysis of cells in ultra-pure H_2_O, followed by addition of Hoechst 33258 (ThermoFisher, #62249) at 1 μg/mL in tris-NaCl buffer (10 mM tris, 2 M NaCl; pH 7.4) at equivalent volume to lysate. Fluorescence (360 nm ex/460 nm em) was measured on a Bio-Tek (Winooski, VA, USA) Synergy 2 microplate reader.

### 4.6. Quantitative PCR Analyses

RNA extractions were performed using the RNeasy Mini kit (Qiagen, Germantown, MD, USA), mRNA was converted to cDNA on an Eppendorf Mastercycler Pro (Eppendorf, Hamburg, Germany) and using Promega (Madison, WI, USA) reagents: oligo (dT)_15_ primer (cat# C110A), random primers (cat# C118A), GoScript 5× reaction buffer (cat# A500D), 25mM MgCl2 (cat# A351H), 10mM dNTPs (cat# U1511), RNasin plus RNase inhibitor (cat# N261B) and GoScript reverse transcriptase (cat# A501D). qPCR reactions were performed with PowerUp SYBR Green Master Mix (Life Technologies/ThermoFisher, cat #100029284) on a QuantStudio 6 Flex real-time PCR system (ThermoFisher). Expression data were normalized to *RPLP0*. Primer sequences were published previously [14,20].

### 4.7. Metabolic Analyses

For analysis of ATP per cell, cells were reverse transfected as above, and at the indicated timepoint, parallel plates were assessed for total dsDNA (above) and total ATP (Cell Titer-Glo, Promega). Cell number by each method was normalized to a standard curve, and analyzed as a ratio of (cell number by ATP)/(cell number by dsDNA).

### 4.8. Transmission Electron Microscopy and Mitochondrial Phenotype Analysis

MM134 cells were reverse transfected with siRNA as above, and 48 h later plated in technical duplicate per condition to 6 cm plates. Then, 24 h later (i.e., 72 h post-transfection), cells were collected by saline-EDTA wash and scraping, then fixed and pelleted in 2% glutaraldehyde, further processing was performed by the U. Colorado Anschutz Electron Microscopy Center. Pellets were re-suspended in 5% agarose that was allowed to harden, and then cut into small pieces for processing. Embedded cells were first rinsed three times in 0.1 M sodium cacodylate buffer (pH 7.4), then post-fixed in 1% osmium tetroxide for 1 h. After three washes in water, the cells were stained en bloc with 2% uranyl acetate for 1 h at 4 °C. Following dehydration through a graded ethanol series (50%, 75%, 95%, 100%) and propylene oxide for 10 min each, the samples were infiltrated with LX112 resin. The samples were embedded and cured for 48 h at 60 °C in an oven. Ultra-thin sections (60 nm) were cut on a Reichert Ultracut S (Leica) from a small trapezoid positioned over the cells and were picked up on EMS copper mesh grids. Sections were imaged on a FEI Tecnai G2 transmission electron microscope (Hillsboro, OR, USA) with an AMT digital camera (Woburn, MA, USA). From collected images, mitochondrial dimensions were measured in ImageJ [82] as described by Tobias et al. [83]. Briefly, mitochondrial outer membranes were traced using the freehand selection tool, and then fit to ellipses. Area measurements were taken from the fit ellipses, data from the technical duplicates were combined.

### 4.9. TCGA Data Analyses

TCGA data were accessed via the cBio portal in March–June 2019, provisional datasets were used for all analyses described. Ovarian cancer and ILC data were updated in June 2020 using the Firehose Legacy datasets. Z-score cutoffs for *WNT4* expression (RNAseq) were selected based on the minimum z-score needed to exclude any low *WNT4*-expressing tumors. Statistical analyses were derived from cBio analysis tools, e.g., enrichments > protein > RPPA.

### 4.10. Data Availability

Data associated with experiments herein will be available at an Open Science Framework repository [81] (https://doi.org/10.17605/OSF.IO/7X8NG) upon publication, or upon request.

## 5. Conclusions

In models of invasive lobular carcinoma of the breast (ILC), ER-driven WNT4 signaling is critical for cell proliferation and survival, but downstream signaling mediated by ER:WNT4 is unknown. We identified mTOR signaling, via S6K, and MCL-1 as key targets of ER:WNT4 signaling in ILC cells, and that these targets converge upon control of mitochondrial dynamics and cellular metabolism. This study offers new insight into a novel Wnt signaling pathway in ILC cells, which underlies endocrine response and anti-estrogen resistance, and identifies potential targets to inhibit this pathway as new therapeutic strategies against ILC cells.

## Supplementary Materials

The following are available online at https://www.mdpi.com/2072-6694/12/10/2931/s1: Figure S1: The decrease in soluble histone H3 upon estrogen treatment is a subset of total H3 protein, Figure S2: WNT4 knockdown in ILC cells dysregulates cell proliferation and PI3K-mTOR networks, Figure S3: WNT4 regulates S6K activity via T421/S424 phosphorylation. Figure S4: ER regulation of MCL-1 is not linked to shifts in sensitivity to BH3 mimetic drugs. Figure S5: RPPA identifies diverse signaling pathways activated during anti-estrogen resistance in ILC models. Figure S6: WNT4 regulation of key targets is consistent across ILC:LTED cell lines. Document S1 (FOXM1); Document S2 (RPPA QC information); Document S3 (Expanded immunoblots); File S1 (RPPA data); Table S1 (TCGA RPPA WNT4 associations); Table S2 (RPPA antibodies).
