# Supplementary material for: Estrogen Regulation of mTOR Signaling and Mitochondrial Function in Invasive Lobular Carcinoma Cell Lines Requires WNT4
Source: Cancers (Basel). 2020 Oct 12;12(10):2931. doi: 10.3390/cancers12102931 (PMC7650584; doi:10.3390/cancers12102931)
Supplement: Supplementary file 1 [file cancers-12-02931-s001.zip › Supplemental Document 1 - FOXM1.pdf]

Supplemental document - FOXM1 is not a direct target of WNT4 signaling

Results

FOXM1 is a critical mediator of estrogen-driven cell cycle progression [1], has been linked to control of ER-driven transcription in IDC models, and is a potential driver of anti-estrogen resistance [1–3]. This led us to examine whether FOXM1 is a critical WNT4 signaling target. We confirmed that *FOXM1* siRNA suppressed the proliferation of MM134 cells (**Figure Panel A**). However, this was not equivalent to that observed with anti-estrogens or siWNT4. Whereas siWNT4 parallels inhibition of ER with fulvestrant and causes a G1 arrest, siFOXM1 causes a G2/M arrest (**Figure Panels B-C**), consistent with established roles of FOXM1 in progression through mitosis [4]. This suggests decreased FOXM1 levels upon WNT4 knockdown may be secondary to siWNT4-induced G1 arrest, rather than identifying FOXM1 as a direct WNT4 target. To confirm this, we examined whether FOXM1 levels could be restored by rescuing cell proliferation via CDKN1A/p21 knockdown, which partially restores proliferation after siWNT4 in ILC cells [5]. Since p21 knockdown partially releases siWNT4-induced G1 arrest, FOXM1 levels should increase as cells progress through G2/M despite WNT4 knockdown. In both MM134 and 44PE, siCDKN1A partially restored the FOXM1 decrease caused by siWNT4 (**Figure Panel D**), supporting that FOXM1 protein level is acting as a marker of cell cycle and G2/M progression. These data suggest that FOXM1 is not a direct target of ER and WNT4 in ILC but is an indirect output of ER:WNT4-driven cell cycle progression.

Supplemental Methods

For cell cycle analyses, RNAi was performed by siRNA transfection as described in the main text, using additional construct pools targeting Human *FOXM1* (M-00976200-0005) and Human *CDKN1A* (M-003471-00-0005). 48 hours after siRNA transfection, or drug treatment, cells were fixed in 70% EtOH overnight at 4°C. After fixation, cells were treated with RNase A, and stained

with 50μg/mL propidium iodide + 0.1% Triton X-100 in PBS overnight at 4°C. PI intensity was assessed on a Gallios Flow Cytometer (Beckman Coulter) at the U. Colorado Cancer Center Flow Cytometry Core Facility; at least 10,000 events were counted per sample.

Supplemental References

1. Millour J, Constantinidou D, Stavropoulou a V, Wilson MSC, Myatt SS, Kwok JM-M, et al. FOXM1 is a transcriptional target of ERalpha and has a critical role in breast cancer endocrine sensitivity and resistance. Oncogene. Nature Publishing Group; 2010 May 20;29(20):2983–95. PMID: 20208560
2. Sanders DA, Ross-Innes CS, Beraldi D, Carroll JS, Balasubramanian S. Genome-wide mapping of FOXM1 binding reveals co binding with estrogen receptor alpha in breast cancer cells. Genome Biol. 2013 Jan 24;14(1):R6. PMID: 23347430
3. Bergamaschi A, Madak-Erdogan Z, Kim YJ, Choi Y-L, Lu H, Katzenellenbogen BS. The forkhead transcription factor FOXM1 promotes endocrine resistance and invasiveness in estrogen receptor-positive breast cancer by expansion of stem-like cancer cells. Breast Cancer Res. 2014;16(5):436. PMID: 25213081
4. Gormally M V, Dexheimer TS, Marsico G, Sanders D a, Lowe C, Matak-Vinković D, et al. Suppression of the FOXM1 transcriptional programme via novel small molecule inhibition. Nat Commun. 2014;5:5165. PMID: 25387393
5. Sikora MJ, Jacobsen BM, Levine K, Chen J, Davidson NE, Lee A V, et al. WNT4 mediates estrogen receptor signaling and endocrine resistance in invasive lobular carcinoma cell lines. Breast Cancer Res. Breast Cancer Research; 2016 Sep 20;18(1):92. PMID: 27650553

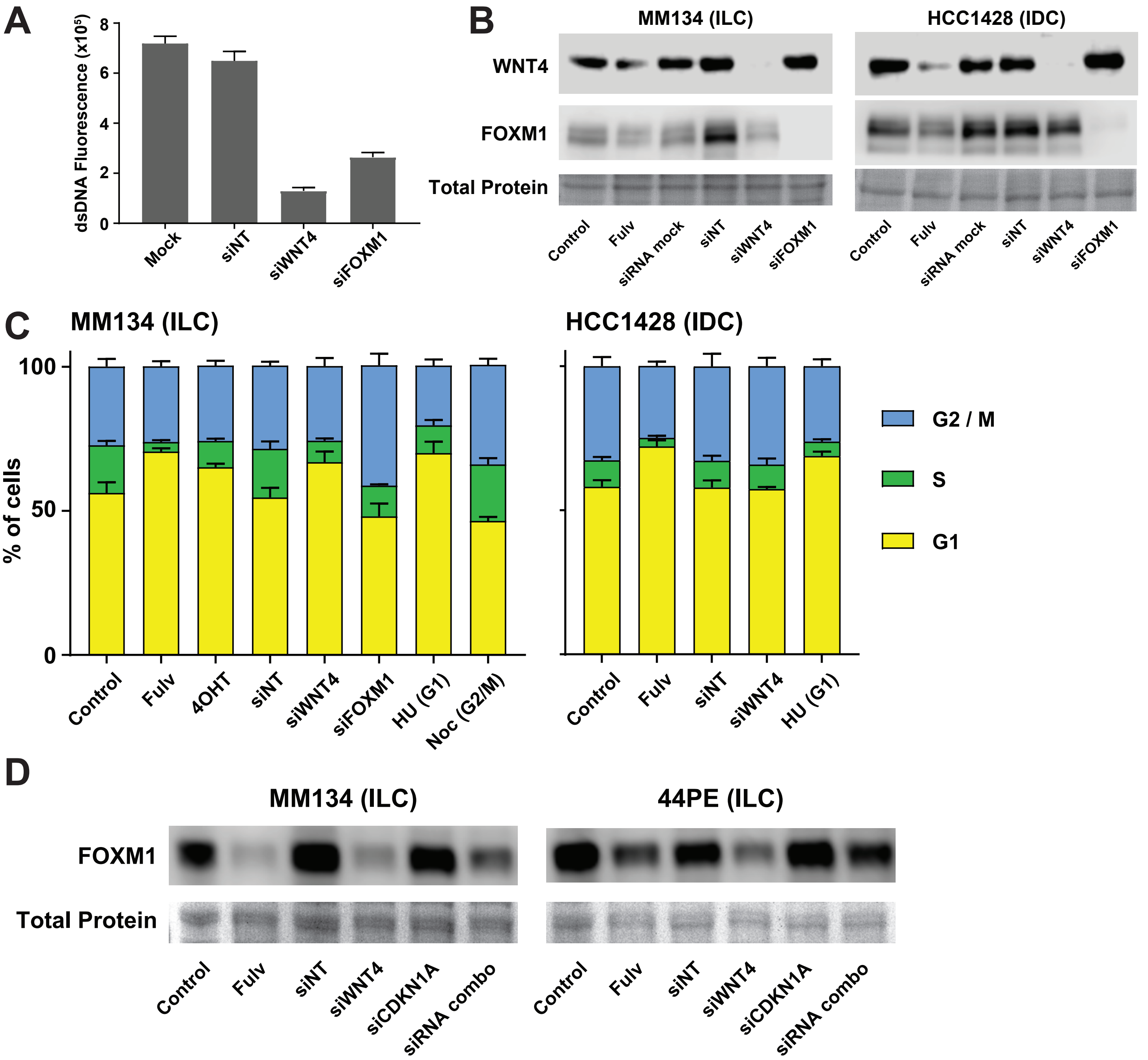

Supplemental Document Figure. FOXM1 is not a direct target of WNT4 signaling. (A), MM134 cells in full serum were reverse transfected with the indicated siRNA, and proliferation was assessed by dsDNA quantification after 6d. Bars represent mean of 6 biological replicates ±SD. (B), Confirmation of WNT4 or FOXM1 knockdown. MM134 cells in full serum were reverse tranfected with the indicated siRNA. 24hrs later, cells were treated with Fulvestrant, and lysates were harvested an additional 24hrs later. Total protein detected with Ponceau staining. (C), Cells in full serum were transfected and treated as in (B) using 1μM Fulvestrant, 1μ M 4-hydroxytamoxifen, 2mM hydroxyurea, or 50ng/mL nodocazole. Bars represent mean cell fraction per cell cycle phase of 3 biological replicate experiments ±SD. (D), Cells were transfected and treated as in (B). In siRNA combinations, siNT were used to increase total siRNA to 20nM in single siRNA samples. Total protein detected with Ponceau staining.
