# Supplementary material for: Estrogen Regulation of mTOR Signaling and Mitochondrial Function in Invasive Lobular Carcinoma Cell Lines Requires WNT4
Source: Cancers (Basel). 2020 Oct 12;12(10):2931. doi: 10.3390/cancers12102931 (PMC7650584; doi:10.3390/cancers12102931)
Supplement: Supplementary file 1 [file cancers-12-02931-s001.zip › Supplemental Document 3 - Expanded Imunoblots.pdf]

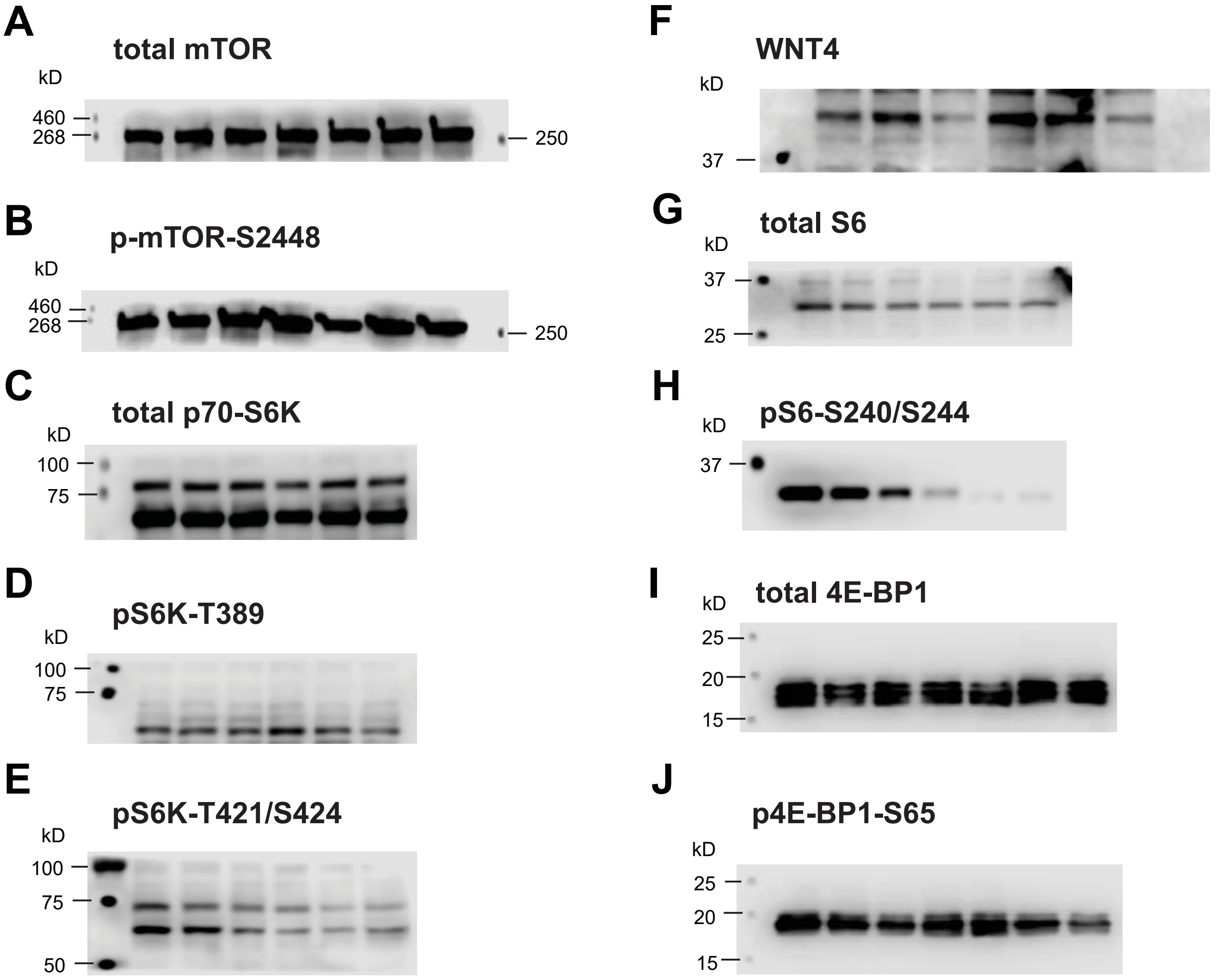

Supplemental Document 2. Molecular weight markers for immunoblots.
Immunoblot images above are derived from figures herein (as noted for each sub-panel) and are representative of all related images. Dots that are labeled as weight markers are from marking the molecular weight ladder with a WesternSure pen prior to imaging. Antibodies are listed in Materials and Methods. (A), Figure S3A - mTOR; predicted weight ~289kD. (B), Figure S3A - p-mTOR-S2488; predicted weight ~ 289kD. (C), Figure 3A - total p70-S6K; predicted weight ~ 70, 85kD. (D), Figure 3B - pS6K-T389; predicted weight ~ 70, 85kD. (E), Figure 3B - pS6K-T421/S424; predicted weight ~ 70, 85kD. (F), Figure S3A - WNT4; predicted weight ~ 39kD. (G), Figure 3A - total S6; predicted weight ~32kD. (H), Figure 3A - pS6-S240/S244; predicted weight ~ 32kD. pS6-S235/S236 also has a predicted weight of ~ 32kD. (I), Figure S3A - total 4E-BP1, predicted weight ~ 15 - 20kD. (J), Figure S3A - p4E-BP1-S65; predicted weight ~ 15 - 20kD. Exposure was increased in some images versus main figures to show ladder markers.
