# Supplementary material for: Estrogen Regulation of mTOR Signaling and Mitochondrial Function in Invasive Lobular Carcinoma Cell Lines Requires WNT4
Source: Cancers (Basel). 2020 Oct 12;12(10):2931. doi: 10.3390/cancers12102931 (PMC7650584; doi:10.3390/cancers12102931)
Supplement: Supplementary file 1 [file cancers-12-02931-s001.zip › Supplemental Figures 1-6 (revision).pdf]

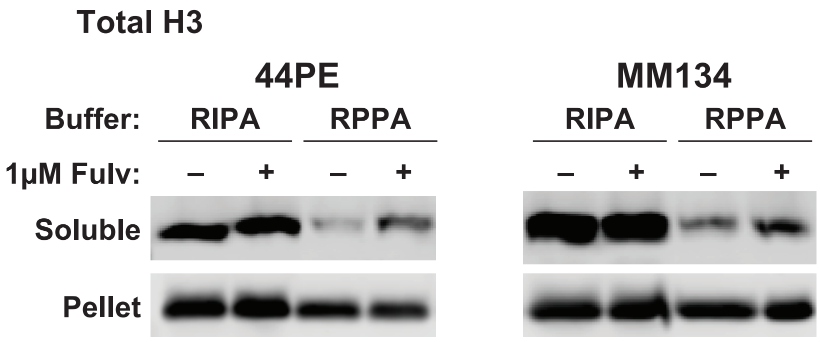

Supplemental Figure 1. The decrease in soluble Histone H3 upon estrogen treatment is a subset of total H3 protein. RPPA analyses in Figure 1 showed that estrogen treatment suppressed total histone H3. To examine this further, we used the reciprocal approach, inhibiting ER with Fulvestrant for 24hrs. Cells lysed with RPPA buffer (lacking SDS) showed the expected increase in H3 upon ER inhibition, but this was not observed in cells lysed with RIPA buffer (containing SDS).

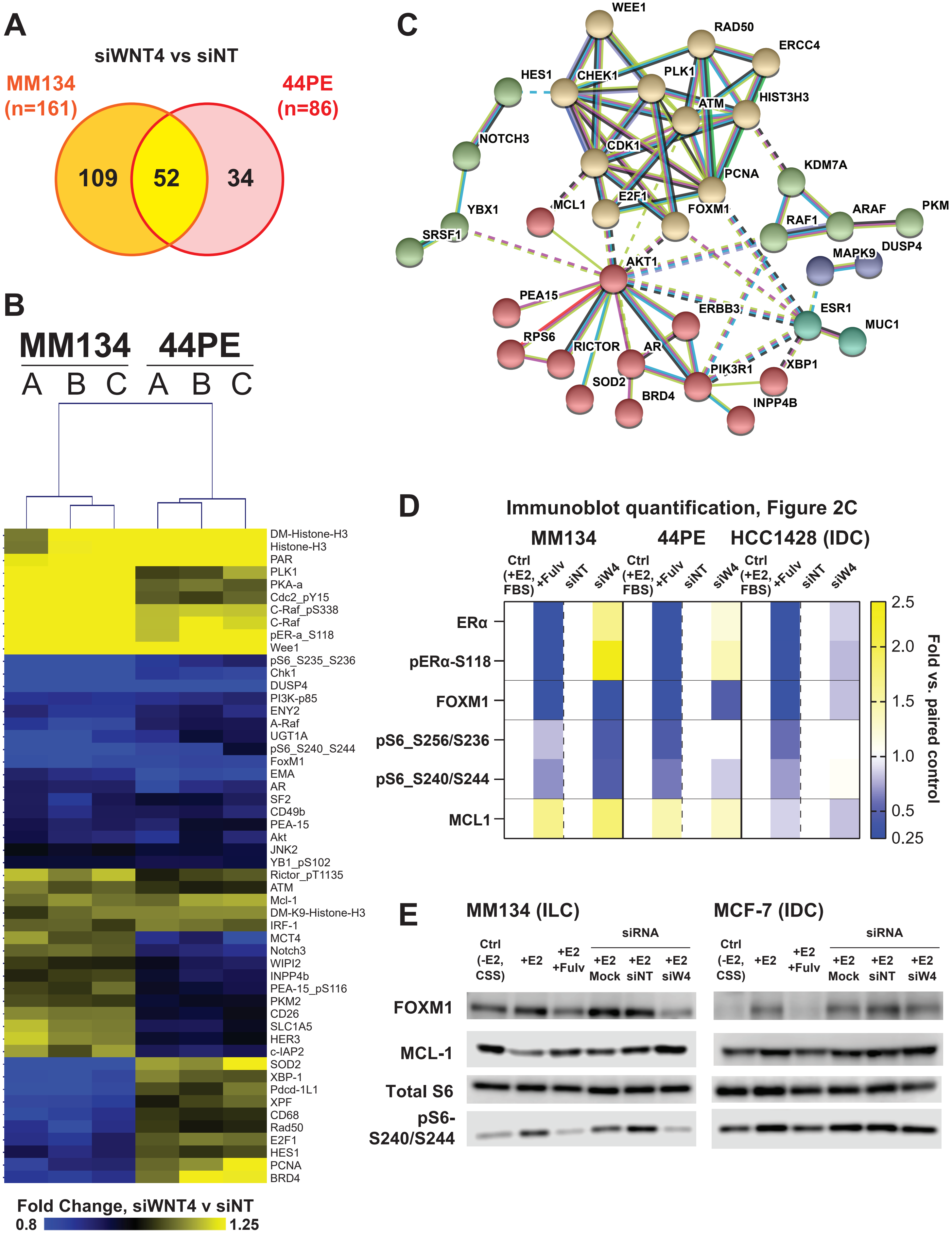
A
siWNT4 vs siNT
MM134
(n=161)
44PE
(n=86)
109
52
34
B
MM134
44PE
A B C A B C
DM-Histone-H3
Histone-H3
PAR
PLK1
PKA-a
Cdc2_pY15
C-Raf_pS338
C-Raf
pER-a_S118
Wee1
pS6_S235_S236
Chk1
DUSP4
PI3K-p85
ENY2
A-Raf
UGT1A
pS6_S240_S244
FoxM1
EMA
AR
SF2
CD49b
PEA-15
Akt
JNK2
YB1_pS102
Rictor_pT1135
ATM
Mcl-1
DM-K9-Histone-H3
IRF-1
MCT4
Notch3
WIPI2
INPP4b
PEA-15_pS116
PKM2
CD26
SLC1A5
HER3
c-IAP2
SOD2
XBP-1
Pdcd-1L1
XPF
CD68
Rad50
E2F1
HES1
PCNA
BRD4
Fold Change, siWNT4 v siNT
0.8
1.25
C
WEE1
RAD50
ERCC4
HES1
CHEK1
PLK1
HIST3H3
ATM
NOTCH3
CDK1
PCNA
KDM7A
MCL1
E2F1
FOXM1
YBX1
SRSF1
RAF1
ARAF
PKM
MAPK9
DUSP4
AKT1
PEA15
ERBB3
ESR1
MUC1
RPS6
RICTOR
AR
XBP1
PIK3R1
SOD2
BRD4
INPP4B
D
Immunoblot quantification, Figure 2C
MM134
44PE
HCC1428 (IDC)
Ctrl (+E2, FBS)
+Fulv
siNT
siW4
ERα
pERα-S118
FOXM1
pS6_S256/S236
pS6_S240/S244
MCL1
2.5
2.0
1.5
1.0
0.5
0.25
Fold vs. paired control
E
MM134 (ILC)
MCF-7 (IDC)
Ctrl (-E2, CSS)
+E2
+E2 +Fulv
siRNA
+E2 Mock
+E2 siNT
+E2 siW4
FOXM1
MCL-1
Total S6
pS6-S240/S244

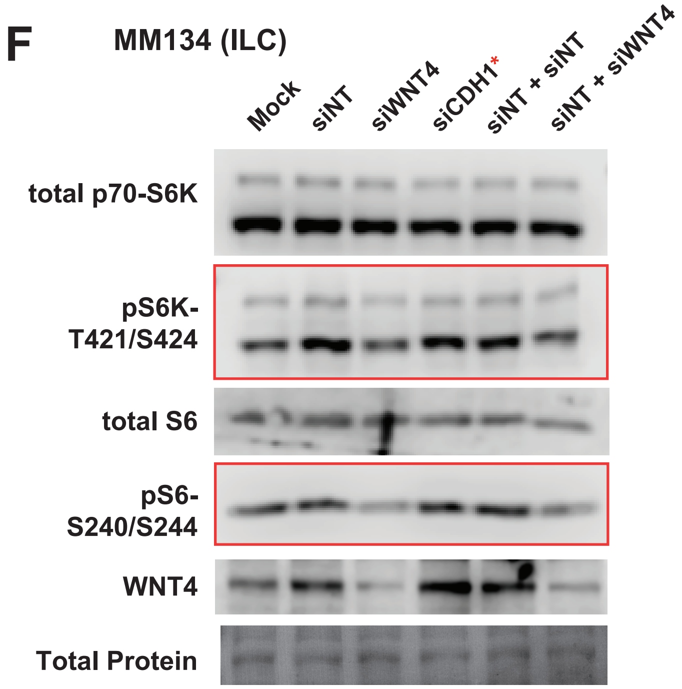

Supplemental Figure 2. WNT4 knockdown in ILC cells dysregulates cell proliferation and PI3K-mTOR networks. (A), Cells were hormone-deprived prior to siRNA reverse transfection and subsequent treatment with 100pM E2 for 24hrs (biological triplicate; see Materials and Methods). RPPA protein signaling changes for siWNT4+E2 versus siNT+E2 shown ($p<0.05$). (B), Heatmap for shared targets, siWNT4 vs siNT. A/B/C represent biological replicate samples. (C), STRING network analysis of WNT4 targets shared in 44PE and MM134 (n=52). Related clusters identified in STRING by MCL clustering are indicated by colored circles, including a PI3K-mTOR cluster (red) and a cell cycle progression/checkpoint cluster (tan). (D), Quantification of immunoblot data in Figure 2C versus total protein. (E), MM134 or MCF-7 were hormone-deprived prior to drug treatment and/or siRNA transfection as indicated (i.e. as performed for RPPA studies). (F), MM134 cells in full serum were reverse transfected with the indicated siRNA for 72hrs. siCDH1 (targeting E-cadherin) was used as an additional "non-targeting" construct, since ILC cells are negative for E-cadherin protein. The red boxes highlight where both siNT and siCDH1 increase mTOR target phosphorylation, indicating a class effect of siRNAs. Combining siWNT4 with siNT blocked NT-driven signaling changes (equivalent total siRNA transfected; 2x siNT vs siNT + siWNT4) This confirms that WNT4 knockdown suppresses mTOR signaling targets and is unlikely an artifact of the NT effect. Total protein detected with Ponceau staining.

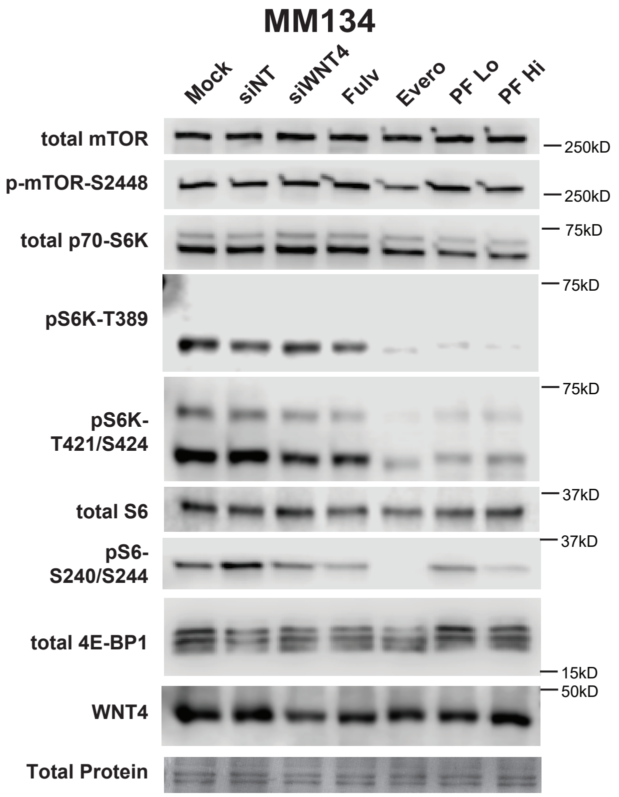

Supplemental Figure 3. WNT4 regulates S6K activity via T421/S424 phosphorylation. MM134 cells in full serum were transfected with siRNA or treated with inhibitors Fulvestrant (Fulv, 100nM), Everolimus (Evero, 10nM, or PF-4708671 (PF, 10μM or 30μM). Cells were harvested 72hrs post-transfection or -treatment, and immunoblotting was performed for mTOR signaling targets as indicated. Ponceau staining shown as total protein loading control.

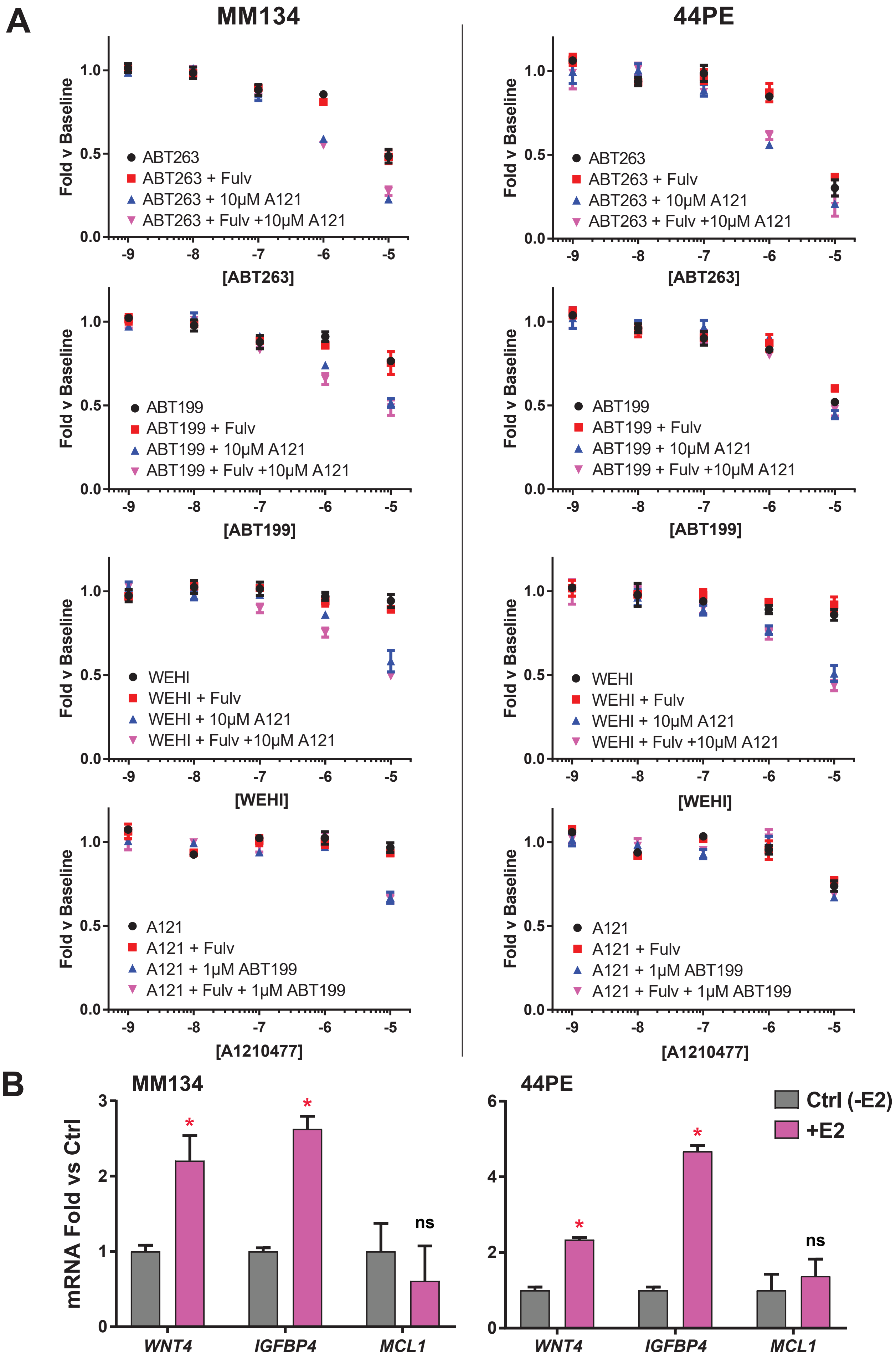
A
MM134
44PE
Fold v Baseline
ABT263
ABT263 + Fulv
ABT263 + 10μM A121
ABT263 + Fulv +10μM A121
[ABT263]
ABT199
ABT199 + Fulv
ABT199 + 10μM A121
ABT199 + Fulv +10μM A121
[ABT199]
WEHI
WEHI + Fulv
WEHI + 10μM A121
WEHI + Fulv +10μM A121
[WEHI]
A121
A121 + Fulv
A121 + 1μM ABT199
A121 + Fulv + 1μM ABT199
[A1210477]
B
MM134
44PE
mRNA Fold vs Ctrl
Ctrl (-E2)
+E2
ns
WNT4
IGFBP4
MCL1

Supplemental Figure 4. ER regulation of MCL-1 is not linked to shifts in sensitivity to BH3 mimetic drugs. (A), Cells in full serum were pre-treated with 100nM Fulvestrant and/or base BH3 mimetic (ie. the compound at static concentration per assay) for 6hrs prior to treatment with increasing concentrations of the test BH3 mimetic. Cell proliferation/death was assessed by dsDNA quantification 72hrs after second treatment. Points represent mean of biological triplicate ±SD. (B), mRNA expression of *MCL1* and ER target genes *WNT4* and *IGFBP4*. Cells were hormone-deprived prior to siRNA reverse transfection and subsequent treatment with 100pM E2 for 24hrs. Expression was normalized to *RPLP0*. *, Ctrl vs +E2, $p < 0.05$, Student's T-test.

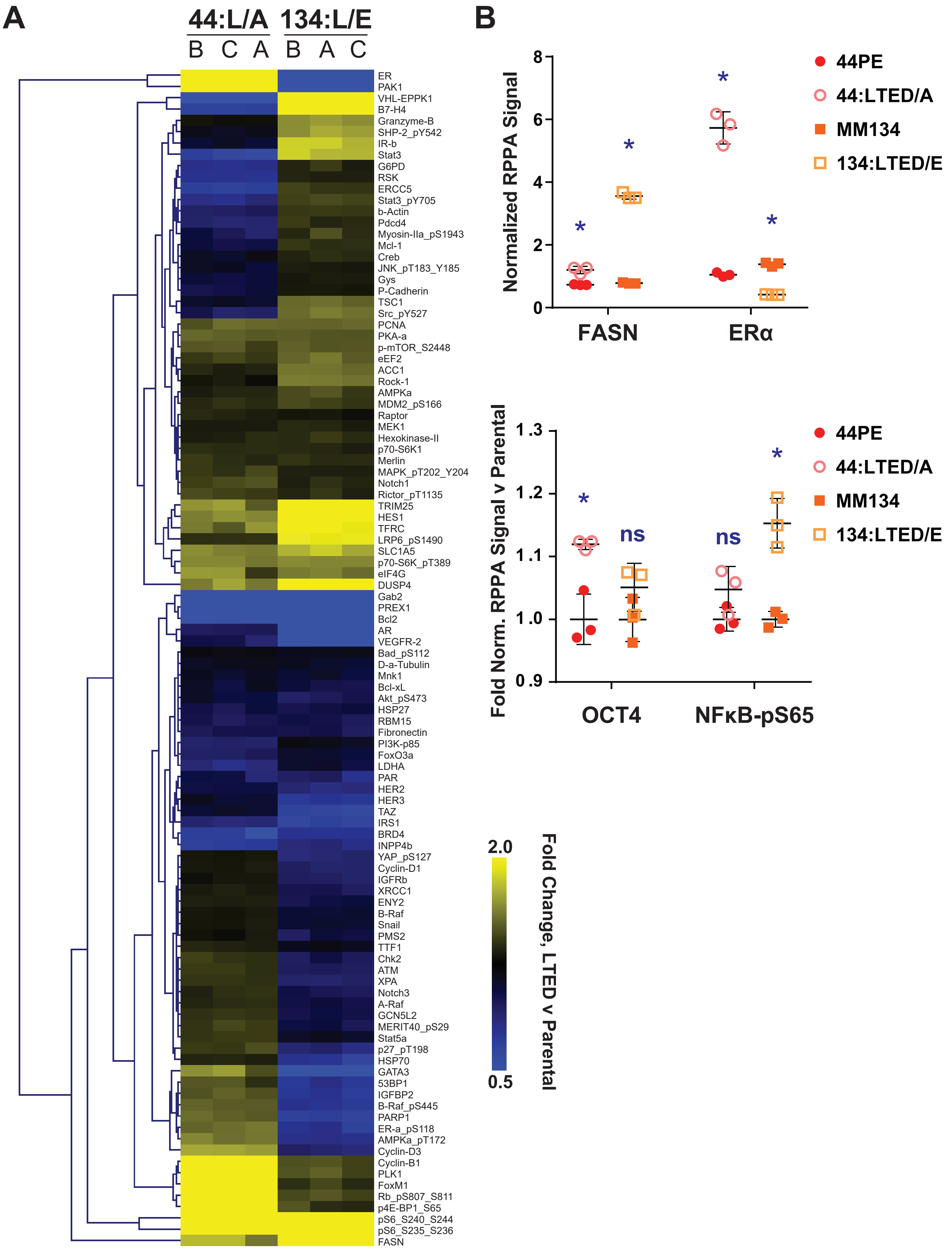
A
44:L/A
134:L/E
B C A
B A C
ER
PAK1
VHL-EPPK1
B7-H4
Granzyme-B
SHP-2_pY542
IR-b
Stat3
G6PD
RSK
ERCC5
Stat3_pY705
b-Actin
Pdcd4
Myosin-IIa_pS1943
Mcl-1
Creb
JNK_pT183_Y185
Gys
P-Cadherin
TSC1
Src_pY527
PCNA
PKA-a
p-mTOR_S2448
eEF2
ACC1
Rock-1
AMPKa
MDM2_pS166
Raptor
MEK1
Hexokinase-II
p70-S6K1
Merlin
MAPK_pT202_Y204
Notch1
Rictor_pT1135
TRIM25
HES1
TFRC
LRP6_pS1490
SLC1A5
p70-S6K_pT389
eIF4G
DUSP4
Gab2
PREX1
Bcl2
AR
VEGFR-2
Bad_pS112
D-a-Tubulin
Mnk1
Bcl-xL
Akt_pS473
HSP27
RBM15
Fibronectin
PI3K-p85
FoxO3a
LDHA
PAR
HER2
HER3
TAZ
IRS1
BRD4
INPP4b
YAP_pS127
Cyclin-D1
IGFRb
XRCC1
ENY2
B-Raf
Snail
PMS2
TTF1
Chk2
ATM
XPA
Notch3
A-Raf
GCN5L2
MERIT40_pS29
Stat5a
p27_pT198
HSP70
GATA3
53BP1
IGFBP2
B-Raf_pS445
PARP1
ER-a_pS118
AMPKa_pT172
Cyclin-D3
Cyclin-B1
PLK1
FoxM1
Rb_pS807_S811
p4E-BP1_S65
pS6_S240_S244
pS6_S235_S236
FASN
2.0
0.5
Fold Change, LTED v Parental
B
Normalized RPPA Signal
8
6
4
2
0
FASN
ERα
44PE
44:LTED/A
MM134
134:LTED/E
Fold Norm. RPPA Signal v Parental
1.3
1.2
1.1
1.0
0.9
ns
OCT4
NFκB-pS65

Supplemental Figure 5. RPPA identifies diverse signaling pathways activated during anti-estrogen resistance in ILC models. (A), Heatmap for n=104 shared protein signaling changes for LTED versus parental cells. A/B/C represent biological replicate samples. (B), Protein changes in LTED models that support previously described features of the anti-estrogen resistance phenotype in these models. Points represent individual biological replicates. *, Matched parental v LTED, q<0.05.

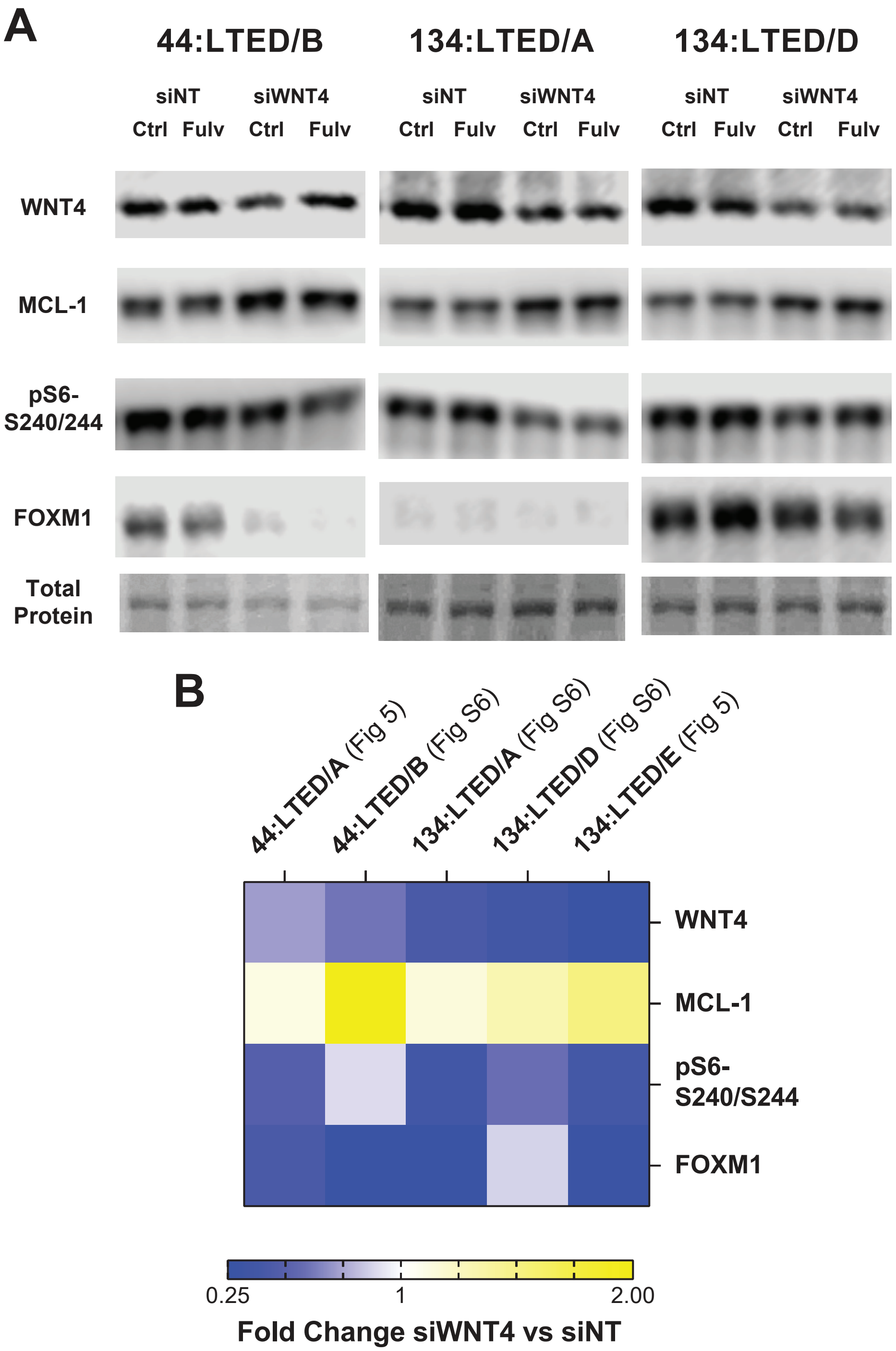

Supplemental Figure 6. WNT4 regulation of key targets is consistent across ILC:LTED cell lines. (A), LTED cells (in hormone-deprivation) were reverse transfected with the indicated siRNA. 24hrs later, cells were treated with 1µM Fulvestrant or vehicle, and lysates were harvested after an additional 24hrs. Total protein detected with Ponceau staining. (B), Quantification (normalized to total protein) for siWNT4 vs siNT for LTED blots in Figure 5 and Supplemental Figure 6A.
